# Two routes to actorhood: lexicalized potency to act and identification of the actor role

**DOI:** 10.3389/fpsyg.2015.00001

**Published:** 2015-01-30

**Authors:** Sabine Frenzel, Matthias Schlesewsky, Ina Bornkessel-Schlesewsky

**Affiliations:** ^1^Department of Germanic Linguistics, University of MarburgMarburg, Germany; ^2^Department of English and Linguistics, Johannes Gutenberg University MainzMainz, Germany; ^3^Cognitive Neuroscience Laboratory, School of Psychology, Social Work and Social Policy, University of South AustraliaAdelaide, SA, Australia

**Keywords:** language comprehension, actor, causality, agency, event-related potentials, N400, extended argument dependency model

## Abstract

The inference of causality is a crucial cognitive ability and language processing is no exception: recent research suggests that, across different languages, the human language comprehension system attempts to identify the primary causer of the state of affairs described (the “actor”) quickly and unambiguously (Bornkessel-Schlesewsky and Schlesewsky, 2009). This identification can take place verb-independently based on certain prominence cues (e.g., case, word order, animacy). Here, we present two experiments demonstrating that actor potential is also encoded at the level of individual nouns (a *king* is a better actor than a *beggar*). Experiment 1 collected ratings for 180 German nouns on 12 scales defined by adjective oppositions and deemed relevant for actorhood potential. By means of structural equation modeling, an actor potential (ACT) value was calculated for each noun. Experiment 2, an event-related potential study, embedded nouns from Experiment 1 in verb-final sentences, in which they were either actors or non-actors. N400 amplitude increased with decreasing ACT values and this modulation was larger for highly frequent nouns and for actor versus non-actor nouns. We argue that potency to act is lexically encoded for individual nouns and, since it modulates the N400 even for non-actor participants, it should be viewed as a property that modulates ease of lexical access (akin, for example, to lexical frequency). We conclude that two separate dimensions of actorhood computation are crucial to language comprehension: an experience-based, lexically encoded (bottom–up) representation of actorhood potential, and a prominence-based, computational mechanism for calculating goodness-of-fit to the actor role in a particular (top–down) sentence context.

## INTRODUCTION

The identification of causal relations is a fundamental property of human cognition. When there is an effect, we feel the need to identify a cause and when two events take place in rapid succession, we tend to understand them as causally connected. Identifying causes is typically synonymous with identifying a causer, i.e., a person or thing responsible for the state of affairs in question. Language processing is no exception: it has long been assumed that the language processing system employs certain strategies for the identification of the “actor.” Note that we use the term actor to refer to the participant primarily responsible for a linguistically expressed event or state of affairs. Thus, while actors are prototypically causers, they need not be, e.g., in the case of Experiencers (cf. Van Valin, 2005). (For a more detailed explanation in a psycholinguistic context, see Bornkessel-Schlesewsky and Schlesewsky, 2009.) For example, Bever (1970) posited that (English) sentences are preferentially analyzed as adhering to the structure “actor action object modifier” and many similar strategies have been proposed in subsequent research (e.g., Ferreira, 1994, 2003; Townsend and Bever, 2001).

Recent research on sentence comprehension has revealed that, beyond these sentence-level heuristics relating actors with actions and patients, the identification of the actor itself seems to be of particular importance. Thus, typologically diverse languages (including English, German, Mandarin Chinese, Tamil), (a) show a tendency for initial arguments to be analyzed as actors whenever possible; and (b) penalize the processing of non-prototypical actors. Both of these observations are independent of verb information as they occur even before the verb is encountered in verb-final sentences (for a detailed discussion of these properties and the findings from different languages, see Bornkessel-Schlesewsky and Schlesewsky, 2009). From findings such as these, we have proposed that linguistic actorhood is based on a “language-independent [actor] category, possibly rooted in the human ability to understand goal-directed action” (Bornkessel-Schlesewsky and Schlesewsky, 2013b) and that this accounts for the cross-linguistic importance of actor identification during language comprehension.

Several previous results indicate that online actor identification may be influenced by fine-grained lexical information in combination with real-world knowledge (e.g., McRae et al., 1997, 1998). For example, McRae et al. (1998) contrasted sentences such as (1) in a self-paced reading study.

(1) Role-filler biases which modulate reading time patterns (McRae et al., 1998, segmentation for self-paced reading indicated by slashes)
    (a) The cop/arrested by/the detective/was guilty/of taking/bribes.
    (b) The crook/arrested by/the detective/was guilty/of taking/bribes.
     (+unambiguous control conditions)

In the sentences in (1), the sentence initial noun phrase (NP) is either a good actor (*cop*, 1a) or a good undergoer (*crook*, 1b) for the following verb (*arrested*) and this goodness of fit (determined via a norming study) was independent of animacy (only animate nouns were used; most were human, some were animals). Reading times showed a strong influence of verb-specific role-filler congruence. At the verb + preposition region, there was a general penalty for reduced relative clauses, but this was larger for the sentences in which the first argument was a good undergoer, i.e., when NP1 and the verb combined to yield an unlikely actor-verb sequence. At the actor NP region, reading times were slowed for reduced relative clauses and for sentences in which the initial NP was a good undergoer rather than a good actor.

The findings by McRae et al. (1998) indicate that the goodness of fit between a noun and the roles specified by the verb is, at least in part, determined by lexical features and that this information appears to be used quite rapidly during the comprehension process (though how immediate its application is cannot be determined by means of self-paced reading). These results thus suggest that actor identification can be understood within the context of a larger body of psycholinguistic work which demonstrates that lexical information plays an important role in incremental language comprehension (e.g., MacDonald et al., 1994; Trueswell and Tanenhaus, 1994; Jurafsky, 1996; Vosse and Kempen, 2000; for a recent overview, see McRae and Matsuki, 2013). From this perspective, actor identification could be viewed as a special case of constraint satisfaction: competition between possible alternative interpretations (role assignments) leads to a particular participant being interpreted as actor (McRae et al., 1998; see also the notion of “competition for the actor role” in Bornkessel-Schlesewsky and Schlesewsky, 2009, 2013a,b). The proposal that the main burden of sentence processing lies on lexical representations and their interaction has recently gained additional support from the domain of event-related potentials (ERPs) brain, with many researchers arguing that the well-known N400 component is best understood as reflecting lexical preactivation (or the lack thereof) rather than aspects of higher-level linguistic computation (e.g., Kutas and Federmeier, 2000; Lau et al., 2008; Brouwer et al., 2012; Stroud and Phillips, 2012). As the cross-linguistic conclusions relating to the actor role that were mentioned above are primarily based on modulations of the N400, these observations could be viewed as further evidence for a lexicalist perspective on actor identification.

However, a notable restriction on lexicalist accounts of semantic role assignments is that, to date, findings such as those by McRae et al. (1997, 1998) have been confined to noun–verb combinations. It is therefore not clear whether and, if so, how they might generalize to a verb-independent, lexicalized actor concept. This is particularly relevant to the processing of verb-final structures, which are widespread throughout the languages of the world (Dryer, 2005), in combination with the well-established notion of incremental interpretation (e.g., Crocker, 1994; Stabler, 1994). Indeed, previous findings suggest that actor participants are identified incrementally even in verb-final structures and thus independently of verb information (e.g., Bornkessel et al., 2003; for evidence regarding other semantic roles, see Kamide et al., 2003). Accordingly, inanimate (i.e., non-prototypical) actors have been shown to elicit processing difficulties prior to the verb (e.g., increased N400 effects in ERP studies: Weckerly and Kutas, 1999; Roehm et al., 2004, 2007; Philipp et al., 2008) and actor prototypicality has also been shown to modulate the processing of verb-final relative clauses (e.g., Mak et al., 2002, 2006; Traxler et al., 2002, 2005; Chen et al., 2006). This indicates that knowledge regarding prototypical actor properties (e.g., animacy) is used to inform verb-independent actor identification. By contrast, it has hitherto not been examined whether this observation extends beyond broad semantic categories such as animacy to more fine-grained lexical-semantic properties. The aim of the present study was to shed light on this possibility. In the following, we discuss how one might extract the relevant meaning dimensions of nouns before going on to describe a model of an individual noun’s “potency to act” (cf. Bornkessel-Schlesewsky et al., 2013) based on a questionnaire study (Experiment 1) and an ERP study designed to test the electrophysiological correlates of this value (Experiment 2).

A systematic attempt to determine dimensions of word (concept) meaning was undertaken by Osgood and colleagues as early as the 1950s (Osgood, 1952; Osgood et al., 1957, 1975). This method, termed the “semantic differential” measures meaning by examining how a concept is mapped “to an experiential continuum, definable by a pair of polar terms” (Osgood, 1952, p. 227) under the assumption that “[a] limited number of such continua can be used to define a semantic space within which the meaning of any concept can be specified” (Osgood, 1952, p. 227). Specifically, Osgood et al. (1957) showed that, cross-culturally, the affective meaning of words can be represented as a three dimensional semantic space consisting of the dimensions Evaluation (positive–negative), Potency (strong–weak), and Activity (active–inactive). The semantic differential appears suited to our purposes of defining the “actor potential” of individual nouns because (a) it has been tested extensively with noun stimuli in a range of languages, leading to the extraction of a small number of stable meaning dimensions, and (b) one of these meaning dimensions is described as the “potency” of a concept and thereby appears related to the notion of actorhood. The assumption that affective meaning is relevant to the concept of agency and, hence, potentially important for actorhood is not new. Especially in motivational and social psychology, a key driver of agency (i.e., initiative, action) is affect (Rolls, 2000; Reeve, 2009). From this perspective, emotional reactions are key to ensuring a certain degree of flexibility in behavior- (“flexibility of behavioral responses to reinforcing stimuli,” Rolls, 2000, p. 179) and allow for the rapid monitoring of salient stimuli (i.e., typically stimuli of high emotional valence; e.g., Blackwood et al., 2000, p. 878). Additionally, there is neurophysiological evidence that emotional valence and arousal not only influence behavior but also the perception of language as reflected in a manipulation of the N400 effect (e.g., Schirmer et al., 2005; Kanske and Kotz, 2007; Bayer et al., 2010; Chwilla et al., 2011).

The connection between Osgood’s affective dimensions and language interpretation is also supported by more recent behavioral results which indicate that the semantic differential dimensions may indeed be suited to shedding light on causal attributions and, by extension, actorhood. Thus, Corrigan (2001, 2002) showed that the causal attributions in a linguistically expressed event are highly dependent on the Evaluation and Potency ratings of the event participants in relation to the verb. Activity, by contrast, did not have an effect. In a questionnaire study, participants were asked to judge causality in simple transitive sentences (see 2 for examples).

(2) Sample stimulus from Corrigan (2001) to test whether causality would most likely be attributed to the event participant congruent with evaluation and potency of the verb:
    The teenager/elder harassed/praised the elder/teenager. Did the teenager/elder cause the event because he is the kind of person that harasses/praises people? (*very unlikely* 1 2 3 4 5 6 7 *very likely*) Did the elder/teenager cause the event because he is the kind of person that people harass/praise? (*very unlikely* 1 2 3 4 5 6 7 *very likely*) Did something else cause the event? (*very unlikely* 1 2 3 4 5 6 7 *very likely*)

In her study, Corrigan (2001) showed that causal attributions were made to the event participant that most closely matched the evaluation and potency of the verb. In order to account for order effects and attribution biases inherent to the verb, each noun was presented in subject and object position and with a different verb from the opposite verb bias class (e.g., subject bias: *harass, pull, change*; object bias: *praise, encourage, protect*). Corrigan showed that the implicit causality bias of an object-biasing verb such as praise can be overridden by the identity of the event participant. According to an initial rating, *praise* is a positively evaluated verb and the likelihood for *the elder* to do something positive (evaluation value: 5.2) is higher than the likelihood for *the teen* to do something positive (evaluation value: 3.91). The results reveal that causality was more often attributed to *the elder* than to *the teenager* even if it occurred in subject position and the verb-inherent bias would predict attributions to the object (Corrigan, 2001, pp. 300–301). These findings were replicated and extended by Corrigan (2002), thus attesting to the stability of the findings: “When the subject and the verb match, perceivers attribute causality to the subject, but when they do not match, they attribute causality to the object” (Corrigan, 2002, p. 379).

Corrigan’s studies examined how Evaluation and Potency ratings can override implicit causality biases of verbs rather than studying their effect on actorhood as encoded via linguistic features such as case marking. Nevertheless, her findings yielded two important results for present purposes: (a) the semantic features of the participants in a linguistically described event influence readers’/listeners’ expectations about the actions that these participants are likely to perform; and (b) the semantic differential dimensions Evaluation and Potency appear to provide an appropriate and robust characterization of the relevant participant-inherent features. Nevertheless, like the findings by McRae et al. (1997, 1998), Corrigan’s observations pertain mainly to noun–verb combinations and thus do not demonstrate verb-independent lexical influences on incremental actor identification.

The present study therefore aimed to examine the effects of individual nouns’ inherent actor potential independently of the verb. To this end, we performed a questionnaire study (Experiment 1) in which nouns were rated on the scales defined by Osgood et al. (1957, 1975) as well as on scales derived from Primus’s (1999) linguistic actor features (see below). From these, we derived an actor potential value for each individual noun using structural equation modeling (SEM). This value was then used to predict the amplitude of ERP effects correlating with actor processing during online sentence comprehension (Experiment 2).

## EXPERIMENT 1: QUESTIONNAIRE STUDY AND STRUCTURAL EQUATION MODEL

Based on the centrality of the actor construct for sentence processing (Bornkessel-Schlesewsky and Schlesewsky, 2009, 2013a,b), we hypothesized that experience about the suitability of individual nouns to fill the actor role may lead to a lexicalization of actorhood potential. If true, we should be able to quantify actorhood potential for individual nouns. To this end, we conducted an online survey, which was analyzed using confirmatory factor analysis (CFA), a special case of a structural equation model. SEM is a type of probabilistic network modeling, which allows us to study causal relationships by means of path relations (see e.g., Grace et al., 2012). Importantly for present purposes, SEM allows for the inclusion of latent variables (such as actorhood potential or Osgood’s affective dimensions). While these variables are hythesized, they cannot be measured directly, but only inferred from relations between measured variables. Thus, based on theory and previous experimental findings a model is set up which defines causal relations between observed and latent variables. The CFA then tests via an estimation algorithm whether or not the causal relations in the model can be upheld with the gathered data.

### IDENTIFICATION OF SUITABLE RATING SCALES

In a first step, we identified relevant scales as the observed variables, indicating the actorhood potential of a noun, the latent variable (cf. **Table 1**). As the most prototypical actor is a human, we included a scale representing consciousness (CON) as a uniquely human trait that reflects our ability to reason as well as a scale representing animacy (ANI). However, recent corpus studies suggest that properties related to humanness or animacy do not suffice in order to derive the full range of actor-based effects in natural language. Examining impersonal passives in several Germanic languages using data from natural discourse, Primus (2011) observed that the classic generalization that impersonal passives are restricted to human/animate agents is too strong. At least in Dutch and German, the presence of a self-organized (goal-directed) activity appears to suffice for an impersonal passive to be possible [see examples 3 and 4, from Primus’s (2011) corpus].

**Table 1:** Scales used in Experiment 1, abbreviated in terms of indicators (observed variables).

Indicator	Scale (German)	English translation	Dimension
GOA	ziellos – zielgerichtet	aimless –goal-directed	Actorhood
VAL	schlecht – gut	bad – good	Evaluation/Arousal
PLE	unangenehm –angenehm	unpleasant – pleasant	
APP	hässlich – schön	ugly – pretty	
POW	machtlos – mächtig	powerless – powerful	Potency
SIZ	klein – groß	small – big	
STR	schwach – stark	weak – strong	
CON	ohne Bewusstsein –mit Bewusstsein	without consciousness –with consciousness	Humanness
ANI	leblos – lebendig	inanimate – animate	
VOL	leise – laut	quiet – loud	Activity
SPE	langsam – schnell	slow – fast	
AGE	alt – jung	old – young

(3) Dutch:
het systeem is gevuld met lucht en wordt als er een sprinkler is gesprongen als gevolg van brand met water gevuld waarna er wordt geblust.
‘The system is filled with air and when a sprinkler has switched on due to fire it is filled with water whereafter there is spritzing.’
(4) German:
Mit einem Schalter am Amaturenbrett kann der Fahrer jederzeit auf Benzinbetrieb umschalten. Wenn der Gasdruck auf einen zu niedrigen Wert sinkt, wird automatisch umgeschaltet.
‘The driver can always switch to petrol by pressing a button on the dashboard. When the gas pressure sinks to a level that is too low, [the system] switches automatically.’

From examples such as these and additional judgment studies, Primus concludes that goal-directedness should be treated as a dimension of actorhood that is independent of humanness:

“[S]elf-organized activity or motion presupposes an own source of energy and an own motor program specialized for the type of event denoted by the predicate. This kind of motion can be performed by inanimates which have their own specialized motor program.” (Primus, 2011, p. 97)

Crucially, goal-directedness in this sense does not imply that the argument performing the action has a specific goal in mind. Rather, its activity is directed toward a particular (physical) goal, which may well have been programmed into the system to fulfill a certain function (e.g., the sprinklers in example 3 or the system for switching the gas pump in example 4). The importance of goal-directedness in inferring agency independently of humanness is also well-established in psychology (Piaget, 1929; Heider and Simmel, 1944; Premack, 1990; Opfer, 2002) and neuroscience (e.g., Schultz et al., 2005). For instance, Opfer (2002) showed adults and children (4–10 years of age) videos of unfamiliar blobs moving either aimlessly or in a goal-directed fashion. His study demonstrated that goal-directedness is a prominent feature of biological motion and a decisive factor for both adults and children in order to identify novel entities as alive. Thus, even though goal-directedness is often correlated with humanness, the two dimensions are in principle independent of one another and contribute independently to linguistic actorhood. In view of these considerations and following Primus (2011), we hypothesize goal-directedness (GOA) to be a direct indicator of actorhood potential rather than an indicator of humanness.

As demonstrated by Corrigan (2001, 2002), the semantic differential dimensions Evaluation and Potency are relevant for the attribution of causality and hence, the identification of an actor. Accordingly, in the context of a verb, Evaluation has a strong effect on causal attributions that can override the verb-inherent bias. Thus, nouns with either a positive or a negative implied valence serve as good actors. As indicators for this dimension, we used three prominent semantic differential scales measuring emotional valence (VAL), pleasance (PLE), and appearance (APP) according to Osgood et al. (1975). Since the polarity of these ratings only became relevant in relation to the verb, we used absolute values of the resulting ratings in order to assess the strength of the emotional valence (arousal) rather than ipolarity. The semantic differential dimension Potency measures how strong, powerful or large a person or activity is. We thus included three prominent Potency scales relating to power (POW), strength (STR), and size (SIZ) in the model (1975). According to Corrigan (2001) there is no evidence that a noun’s Activity affects causal attributions. However, Corrigan (2001, 2002) only used Activity scales to rate how dynamic or energetic the verbs she used in her study were perceived. For the sake of completeness, we therefore also included three scales for the semantic differential dimension Activity measuring sound volume (VOL), speed (SPE) and age (AGE), respectively, in order to examine whether activity serves as an indicator of a noun’s actorhood potential. A summary of the scales used here is given in **Table 1**.

### MATERIALS AND METHODS

#### Participants

A total of 227 participants completed the online questionnaire. Participants were recruited in Mannheim, Munich, and Marburg and received an invitation via e-mail with a link to the questionnaire. This link could only be accessed once in order to control for multiple participation. After completing the questionnaire, participants could sign up for a lottery to win 1 of 22 book vouchers worth 10€ each. 29 participants were excluded from the analysis as they were non-native speakers of German or bilinguals (14), or their rating pattern resembled outliers (15). 198 participants (98 male and 100 female) were included in the analysis. 89% of the participants were between 18 and 29 years of age (18–20: 22%; 21–23: 34%; 24–26: 26%; 27–29: 7%). Only a minority of the participants (11%) were over 30 years old (30–32: 6%; 33–35: 3%; 36–38: 1%; 39–41: 1%). Most of the participants (82%) were undergraduate students at a German university or a university of applied sciences; 16% of the participants held a university degree (Ph.D. or higher: 2%; 3–6 year degrees: 14%).

#### Materials

The stimulus material consisted of 180 German nouns (see Supplementary Materials for a full list): 60 referring to humans (A: e.g., *butcher, father, nun*), 60 to animals (B: e.g., *cat, bird, wasp*) and 60 to inanimates, respectively. Inanimate nouns were subdivided into 30 concrete things (C: e.g., *chair, hammer, pen*) and 30 abstract concepts (D: e.g., *hope, danger, respect*). The nouns were distributed across six online questionnaires so that each participant rated a subset of 30 nouns on 12 semantic differential scales. The order of the scales was randomized for each word.

#### Procedure

After agreeing to participate in the study, participants received a link to the online survey conducted with the open source application LimeSurvey^1^. They were instructed to complete the questionnaire in one run and were not able to save their responses in order to continue the questionnaire at a later time. Each word was presented on the screen with 12 scales in a randomized order. Participants were required to rate the word on each scale before they could proceed to the next word. After they had rated the 30 words, participants answered a few demographic questions about their language skills, age and education.

#### Data analysis

**FIGURE 1:**
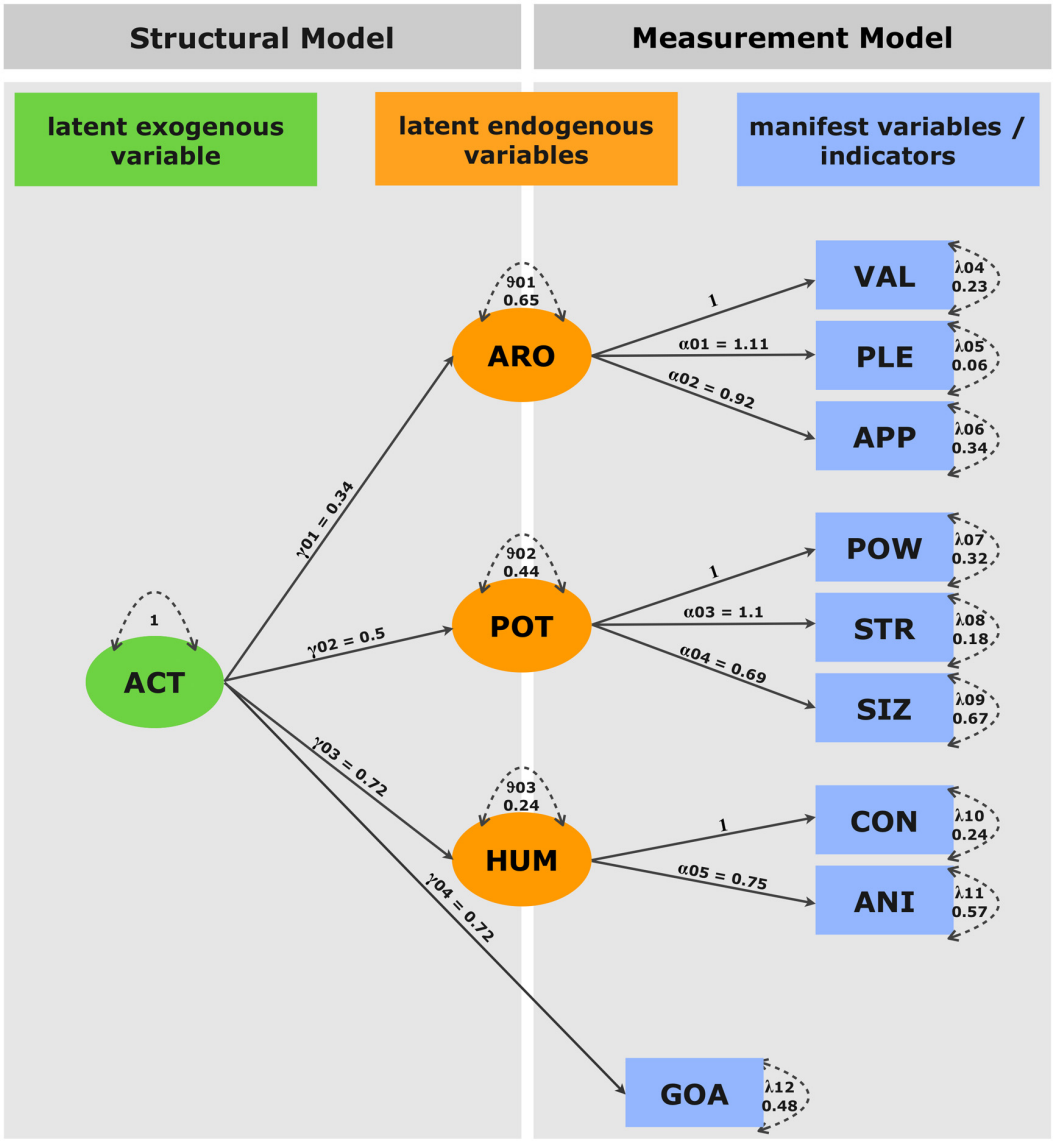
**Structural equation model.** The model consists of a latent exogenous variable (ACT), three latent endogenous variables (EVA, POT, and HUM) and nine manifest variables (VAL, PLE, APP, POW, STR, SIZ, CON, ANI, and GOA). The causal paths gamma 01–04 state that all endogenous latent variables as well as the manifest variable (GOA) are causally dependent upon the exogenous variable (ACT). Alpha 01–05 represent the causal dependency of manifest variables VAL, PLE, and APP on EVA; POW, STR, and SIZ on POT as well as CON and ANI on HUM. Error variance is represented by delta 1–12 (dashed double-headed arrows).

Structural equation modeling was conducted using the sem package (version 0.9-21 in R version 2.11.1) by Fox et al. (2012). The SEM tested here is shown in **Figure 1**, which shows the assumed relations between the manifest (i.e., measurable) and the assumed latent variables. A fundamental assumption of this type of analysis is that correlations between latent and manifest variables and among latent variables are non-zero. Indicators which are not related to a latent variable (factor) are thus considered fixed parameters in the model specified by zero loadings. The goal of a SEM is to find the values of free model parameters, which minimize the discrepancy between the sampled covariance matrix and the model’s reproduced covariance matrix by an iterative algorithm [here: restricted maximum likelihood (REML) estimation].

The three activity-related scales were excluded from the final model because, as in Corrigan’s (2001, 2002) findings, they did not contribute to improving the fit of the model (see below).

### RESULTS

According to the goodness-of-fit-indices in **Table 2**, the comparative fit index (CFI > 0.95), the standardized root mean square residual (SRMR < 0.8) and the root mean square of approximation index (RMSEA < 0.08) suggest a close fit between the sampled data and the estimated model although chi-square is significant on a 0.01 level (Fan et al., 1999; Bühner, 2004). As expected, a model including Activity ratings (**Table 2**, alternative model) did not yield a close fit between sampled data and the estimated model as shown by a highly significant chi-square (*p* < 0.001) and CFI < 0.96.

**Table 2:** Fit indices for structural equation model.

	*p* > χ^2^	DF	RMSEA	SRMR	CFI
	0.004	24	0.068	0.075	0.97
Alternative model	2.6e-06	50	0.078	0.077	0.94

Positive loadings on causal paths (one-headed arrows in **Figure 1**) indicate a positive relation between latent and manifest variables as well as among latent variables. All factor loadings differed significantly from zero (*p* < 0.05). The model depicted in **Figure 1** suggests that an increasing potential to act is positively correlated with arousal (ARO: γ01 = 0.34), potency (POT: γ02 = 0.5), humanness (HUM: γ03 = 0.72), and goal-directedness (GOA: γ04 = 0.72). The same is true for the effect of latent factors on the indicator variables ARO (PLE: α01 = 1.1; APP: α02 = 0.92), POT (STR: α03 = 1.1; SIZ: α04 = 0.69), and HUM (ANI: α05 = 0.75).

In order to compute a value representing the actorhood potential of a given noun (ACT), the ratings per scale were multiplied with their regression coefficients and the products were added (see 5). Error variances were multiplied and subtracted (see the Supplementary Materials for factor values per noun).

(5) ACT = (γ01(ARO – δ01) + γ02(POT – δ02) + γ03(HUM – δ03)) + γ02 ^∗^GOA
    ARO = –1(VAL ^∗^ δ04) + PLE(α01 – δ05) + APP(α02 – δ06)
    POT = –1(POW ^∗^ δ07) + STR(α03 – δ08) + SIZ(α04 – δ09)
    HUM = –1(CON ^∗^ δ10) + ANI(α05 – δ11)

### CROSS-VALIDATION OF RESULTS

In order to examine the validity of the actorhood construct as derived by our structural equation model, we conducted an additional questionnaire study with a total of 67 participants (16 male, 51 female, mean age = 28.5 years). Two participants were excluded because their native language was not German. All participants rated the noun material on a 4-point scale (1 = sehr schlechter Handlungsverursacher ‘very bad actor,’ 2 = eher schlechter Handlungsverursacher ‘rather bad actor,’ 3 = eher guter Handlungsverursacher ‘rather good actor,’ 4 = sehr guter Handlungsverursacher ‘very good actor’). Note that the noun “Handlungsverursacher,” which was used to instruct participants, is more specific than “actor” in English and translates approximately as “event instigator.” Thus, this rating study essentially tested participants’ intuitions regarding relatively prototypical actors.

**FIGURE 2:**
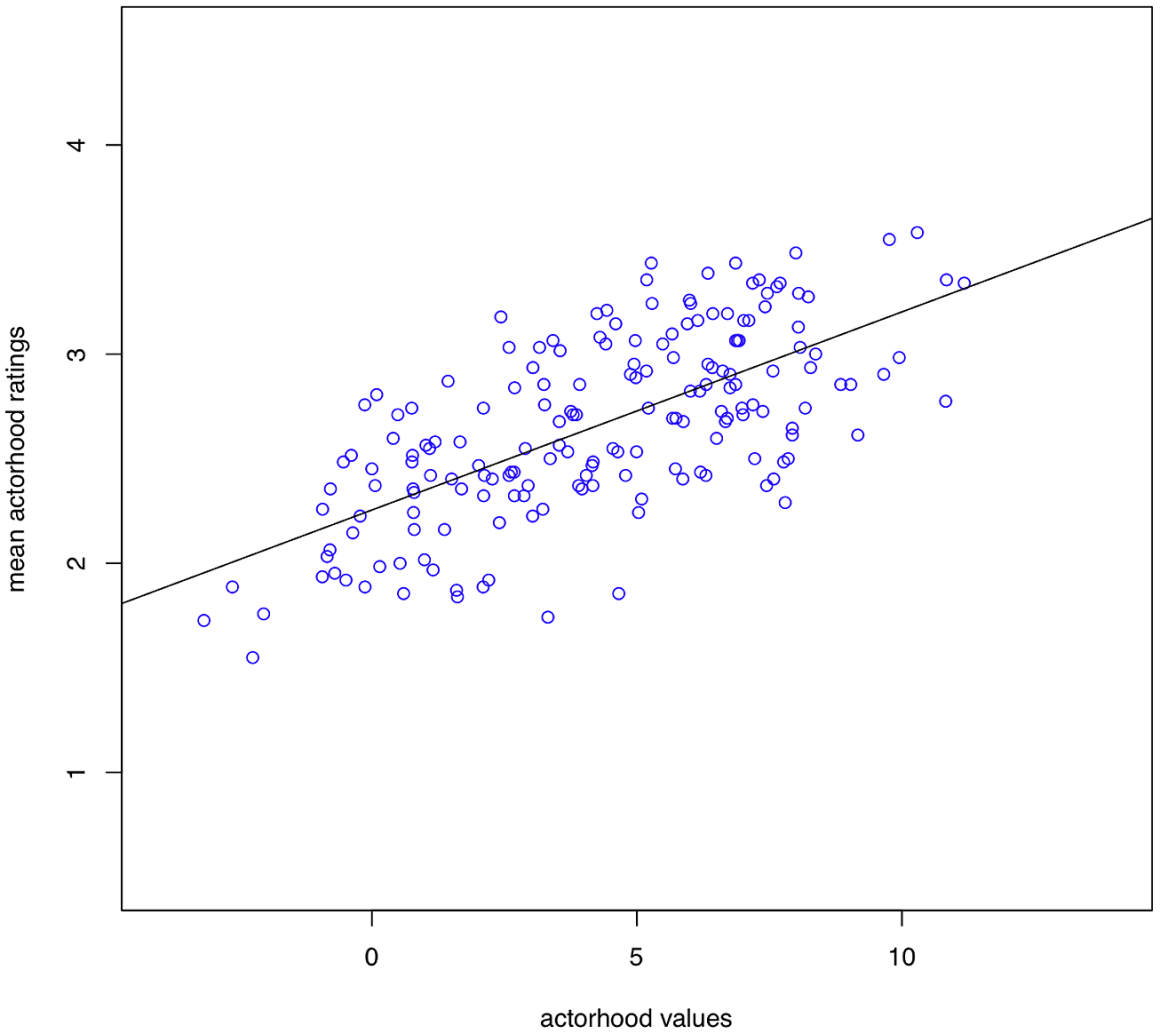
**Visualization of the cross-validation analysis.** Correlation between the actorhood values for individual nouns derived from the structural equation model in **Figure 1** and participants’ ratings of actor goodness in the validation study.

Mean ratings for each noun were compared to the actorhood potential computed according to the structural equation model. A simple correlation of the values revealed a correlation coefficient of 0.67 (*95% confidence interval = 0.58–0.74*), thus demonstrating that our model measures the concept or construct that it is intended to measure (see **Figure 2**). All in all, this result attests to the psychological validity of the “actorhood” concept. Nevertheless, it is subject to the limitation that, since it is very difficult – if not impossible – to instruct naïve participants to rate nouns in accordance with the precise linguistic meaning of actor as a generalized semantic role, the ratings given here were based on the notion of “event instigator” as a relatively prototypical instance of an actor.

### DISCUSSION

The structural equation model constructed on the basis of the data from Experiment 1 suggests that a noun’s actor potential depends on its humanness, its arousal level, its potency and its ability to behave in a goal-directed manner. Thereby, a good actor is conscious and animate, emotionally arousing (positive or negative), perceived as potent (strong, powerful, and big) and moves in a goal-directed manner. This demonstrates that fine-grained semantic characteristics that go beyond the mere animate-inanimate dichotomy influence the goodness of an actor, thus providing a measure to differentiate “good” human actors (e.g., mother, murderer, fighter or doctor) from “poor” human actors (e.g., widow, beggar, pensioner, or servant). Moreover, our findings attest to the psychological validity of our multidimensional actor construct (ACT): ACT shows a high correlation with people’s overt judgments about the goodness-of-actorhood of individual nouns. A parsimonious explanation for this correlation is that people’s judgments about actorhood potential are indeed based on the dimensions contributing to the ACT value. The effects of this actor potential on real time language processing were investigated in Experiment 2.

## EXPERIMENT 2: ERP STUDY

In a second step, we investigated whether the goodness of an actor has an impact on online language processing. We chose ERPs as they provide a fine-grained, multidimensional measure of language processing with a very high temporal resolution (e.g., Kutas et al., 2006). To this end, we used the noun material from Experiment 1 and treated the actor value (ACT) as a parametric variable. In order to analyze the electroencephalography (EEG) data, we used mixed-effects modeling (Baayen, 2008), which, unlike the common ANOVA approach, allows for the inclusion of parametric variables. It also takes variance per participant and stimulus item into account and is thus a very promising approach for the analysis of linguistic ERP data in which by-item variability is an important factor.

In order to measure the effects of individual-noun ACT values during sentence comprehension, we capitalized on previous ERP results on pre-verbal actor identification. A number of studies in several typologically diverse languages have demonstrated that a non-prototypical (inanimate) actor following an undergoer engenders an increased N400 effect in comparison to a prototypical actor. For example, Weckerly and Kutas (1999) observed an N400 effect at the relative clause subject noun for “The editor that the poetry...” in comparison to “The poetry that the editor...” in English and comparable results have been demonstrated for German (Roehm et al., 2004), Mandarin Chinese (Philipp et al., 2008), and Tamil (Muralikrishnan et al., in press). We have argued that this effect can be attributed to the non-fulfillment of a prediction for a prototypical actor which is set up when an undergoer is processed (see Bornkessel and Schlesewsky, 2006; Bornkessel-Schlesewsky and Schlesewsky, 2009, for reviews and discussion). By contrast, undergoer arguments do not engender comparable atypicality effects (see Bornkessel-Schlesewsky and Schlesewsky, 2009; Paczynski and Kuperberg, 2011). If the basic assumptions of lexically based models of sentence comprehension indeed extend to incremental interpretation independently of the verb, lexical actor potential, as defined via a noun’s ACT value, should engender a similar N400 modulation to that elicited by inanimate actors in previous studies. In order to test this hypothesis, we constructed sentences such as those in (6).

(6) Anja fragte sich, ...
     Anja asked herself...
     (a) Subject-initial target sentence (critical NP = undergoer)
          ... wer den Anwalt eingeschaltet hat.
          ... who_NOM_ [the attorney]_ACC_ employed has
          ‘... who employed the attorney.’
     (b) Object-initial target sentence (critical NP = actor)
          ... wen der Anwalt verteidigt hat.
          … who_ACC_ [the attorney]_NOM_ defended has
          ‘... who the attorney defended.’

As is apparent from the sentence conditions in (6), we used embedded wh-questions in order to construct German sentences with a verb-final order, thus allowing us to examine the effects of actor potential independently of verb-based information. (Note that sentences were completed in order to be maximally plausible, as shown by the two different completions following the critical NP, the attorney, in (6), since no ERPs were analyzed at the verb and auxiliary positions.) A second advantage of using wh-questions of the type in (6) (i.e., questions with wh-pronouns) was that they minimized the semantic content of the first NP and kept it constant across all sentences. Thus, wh-pronouns only differed with regard to their case marking, thereby rendering the following critical NP either an undergoer (6a) or an actor (6b). Nouns within the critical NP were taken from the questionnaire study in Experiment 1 and varied with respect to their actor potential (ACT value as derived from the structural equation model).

If the hypothesis advanced above is correct, i.e., if actor potential modulates N400 amplitude for predicted actor participants (i.e., actors following undergoers), we should observe a modulation of the N400 via ACT values for object-initial orders as in (6b). Subject-initial sentences such as (6a) served as controls and as a means of examining the degree of lexicalization of the ACT value. Thus, if the ACT values influence processing in exactly the same way as animacy, we would predict an interaction between ACT and word order (WO), with only object-initial sentences showing an ACT modulation. If, by contrast, ACT affects processing in the same way as an inherent lexical property (e.g., concreteness, frequency), we should observe ACT-based effects for all nouns, i.e., even in subject-initial orders, though perhaps to a lesser degree than in the object-initial sentences.

### MATERIALS AND METHODS

#### Participants

A total of 41 native speakers of German participated in the ERP experiment after giving informed consent (22 women and 19 men; mean age: 25 years, range: 19–32). All participants were right handed and reported normal or corrected to normal vision. One female participant was excluded from the analysis due to software problems.

#### Materials

A total of 168 nouns were used in sentences with subject -initial (SO) and object-initial (OS) WOs (see example 10), thus yielding a total of 336 critical sentences. Each noun’s potential to be a good actor was obtained from the online questionnaire (Experiment 1) in conjunction with the structural equation model as outlined in Section “Experiment 1: Questionnaire Study and Structural Equation Model.” All nouns were two syllables in length (mean length in characters: 6.54; standard deviation: 1.55). The material was divided into two lists of 168 sentences each, which were presented in pseudo-randomized order. Each participant saw only one list of materials. After each sentence, participants responded to a yes/no comprehension question (see **Table 3**). Trials for which the comprehension question was not answered correctly were excluded from further analyses. As is apparent from **Table 3**, several different types of questions were constructed in order to limit participants’ ability to strategically prepare for a particular question type during sentence processing. S-questions asked whether the NP that served as the grammatical subject of the preceding sentence was responsible for the event. These questions always had to be answered with yes in order to be correct. Questions which had to be answered with no varied in order to exclude answering strategies. N-questions asked for an incorrect noun and V-questions for an incorrect verb. O-questions asked whether the NP that served as the grammatical object of the critical sentence was responsible for the event (i.e., reversed thematic role assignments).

**Table 3:** Set of experimental stimuli consisting of a matrix clause ([proper name] asked himself/herself...) and an embedded subordinate clause.

Matrix clause: Anja fragte sich,		Comprehension questions:			
(Anja asked herself, ...)						
	Subordinate clause:	S-question:	N-question:	V-question:	O-question:
SO:	... wer den Anwalt eingeschaltet hat.	Hat jemand den Anwalt eingeschaltet?	Hat jemand den Richter eingeschaltet?	Hat jemand den Anwalt verteidigt?	Hat der Anwalt jemanden eingeschaltet?
	... who the attorney employed has ... ’who employed the attorney.’	Did someone employ the attorney?	Did someone employ the judge?	Did someone defend the attorney?	Did the attorney employ someone?
OS:	... wen der Anwalt verteidigt hat.	Hat der Anwalt jemanden verteidigt?	Hat der Richter jemanden verteidigt?	Hat der Anwalt jemanden eingeschaltet?	Hat jemand den Anwalt verteidigt?
	... whom the attorney defended has ... ’whom the attorney defended.’	Did the attorney defend someone?	Did the judge defend someone?	Did the attorney employ someone?	Did someone defend the attorney?
	Total number of questions	168	56	56	56
	Expected response?	yes (correct)	no (incorrect)		

*Each critical noun phrase (underlined) was presented in subject initial (SO) and object-initial (OS) word order. Only one comprehension question per sentence occurred (in black). Questions in gray represent possible questions and are depicted in order to exemplify stimulus generation only.*

#### Procedure

Participants were seated in front of a computer screen in a sound-proofed booth. Each trial began with the presentation of a fixation cross [400 ms followed by an inter-stimulus-interval (ISI) of 100 ms]. Subsequently, sentences were presented visually in a word-by-word manner, with the exception of critical NPs, which were presented together as a single phrase (e.g., der Anwalt ‘the attorney’). This phrase-by-phrase presentation mode is very common in psycholinguistic and neurolinguistic research on German (e.g., Mecklinger et al., 1995; Friederici et al., 1998; Hopf et al., 1998; Frisch and Schlesewsky, 2001; Bornkessel et al., 2002, 2004b), as it serves to rule out ambiguities that are present when the case-bearing determiner is presented without the following noun (e.g., on its own, the determiner “der” is compatible with either a nominative-masculine-singular, a dative-feminine-singular or a genitive-feminine-plural). In the case of present experiment, however, the unambiguously case-marked initial wh-pronoun already disambiguates whether the second NP should be interpreted as a subject or an object, thus substantially reducing the degree of ambiguity.

Words were presented for 400 ms and phrases for 500 ms followed by an ISI of 100 ms. At the end of a sentence, there were 500 ms of blank screen before the presentation of the comprehension question. Following a participant’s response or after the maximal response time of 2000 ms had run out, a further 1000 ms of blank screen preceded the beginning of the next trial. Participants responded to the comprehension question by pushing one of two push-buttons on a response box. Assignments of “yes” and “no” responses to the left and right button were counterbalanced across participants.

#### Electrophysiological recordings

The EEG was recorded from 64 AgAgCl-electrodes fixed at the scalp with an elastic cap (Electrocap International, Eaton, OH, USA). Electrodes were arranged according to the international 10-10 system and average impedances were kept below 4 kΩ. The electrooculogram (EOG) was monitored by means of electrodes at the outer canthi of each eye as well as above and below the right eye. EEG and EOG signals were recorded using two Twente Medical Systems DC amplifiers at a sampling rate of 500 Hz. The signal was referenced to the left mastoid and re-referenced to linked mastoids oﬄine. A bandpass filter from 0.3 to 20 Hz was applied oﬄine to the raw data in order to exclude slow signal drifts. ERPs were not baseline-corrected (for a detailed motivation, see Wolff et al., 2008; for a direct comparison between baseline-corrected and non-corrected, filtered data, see Choudhary et al., 2009; see also recent guidelines for electroencephalographic research, Keil et al., 2014, as well as recent methodological recommendations for the use of filters in EEG research, Widmann et al., 2014). ERP plots were smoothed with an 8 Hz lowpass filter for display purposes only.

#### Data analysis

For statistical analysis of the ERP data, mixed-effects models were used with subjects and items as crossed random effects (e.g., Baayen, 2008; Baayen et al., 2008). The analysis was performed with the lme4 package (Bates et al., 2011) for R (R Development Core Team, 2012). ERPs were timelocked to the onset of the critical NP (underlined) and analyses were performed on mean amplitudes for a typical N400 time-window (300–500 ms). Parametric variables, which served as fixed effects, were: a noun’s actorhood potential (ACT) and its logarithmic frequency of occurrence (FRQ) according to the online corpus “Wortschatz Lexikon” provided by the University of Leipzig, Germany^2^. In order to ensure that the fixed effects of ACT were not driven by individual differences in the subjects, we included a random-slope for subjects in the analysis. Note that a random-slope of item is not included since the item-specific values of ACT and FRQ consistently yield a high correlation of the slope and the intercept of item random effects for each predictor, respectively (–1.00) indicating that such a model would be overparameterized (see, for example, Baayen, 2008). Additional fixed factors included in the analysis were WO (object-before-subject, OS versus subject-before-object, SO) and case ambiguity (CASE; UNAM = unambiguously case marked masculine nouns; AMB = feminine and neuter nouns ambiguously marked for case). Lateral electrodes were grouped into left-anterior (F7, F5, F3, FT7, FC5, FC3), right-anterior (F8, F6, F4, FT8, FC6, FC4), left-posterior (P7, P5, P3, TP7, CP5, CP3) and right-posterior (P8, P6, P4, TP8, CP6, CP4) regions of interest (ROI). ROI was included into the analysis as a four-level factor. We began by fitting a model with all predictor variables and allowed for maximal interactions. This maximal model was reduced in a stepwise fashion by excluding those fixed effects that did not reach significance at *t* ≥ 2. According to Baayen et al. (2008), for large data sets, an absolute value of the *t*-statistic exceeding two indicates significance at the 5%-level. In order to compute *p*-values for the best-fitting model, we performed Markov chain Monte Carlo simulations with 10,000 samples from the posterior distribution (Baayen et al., 2008) without random correlation parameters. For present purposes, we only report effects and interactions involving the factors ACT and WO in accordance with our hypotheses. A detailed summary of the complete model can be found in the Supplementary Materials. Results are reported including coefficient estimates (CEs) and 95% confidence intervals (CIs).

### RESULTS

#### Comprehension task

Across participants and items, the mean accuracy for the behavioral task was 94.55% (7.06% incorrect and 0.18% timeouts). Thus, participants processed the sentences attentively and understood them. In order to analyze the reaction times and the accuracy of the comprehension task, we used a mixed effects model with the factors WO, actorhood potential (ACT), and frequency (FRQ). In order to account for the variance that is caused by the different question types (QTYPE), we also included this variable as a random factor into the model. This additional random factor is justified as the likelihood ratio test comparing the model with (model 2) or without (model 1) a random factor of question type (see **Table 4**) shows a significantly smaller probability for model 2 both comparing the models for reaction time ratings and the accuracy of the responses (for a detailed description of the procedure cf. Baayen, 2008, p. 253). We refrained from including either by-question type, by-subject, or by-item random slopes for ACT as these analyses yielded a high correlation of slope and intercept (1.00) thus indicating that the model is overparameterized. The analysis only revealed a main effect of WO (CE = –50.56, CI = –67.33 to –34.02, *p* < 0.000). In order to account for the binomial distribution of the comprehension ratings (yes/no), we fit a generalized linear mixed model (Baayen, 2008). Unlike in the linear mixed-effects models fit for reaction times and ERP data, the standard error (SE) rather than a CI is reported. We found an interaction of WO and FRQ (CE = 0.22, SE = 0.01, *p* < 0.002) and a three-way interaction of WO, ACT, and FRQ (CE = –0.03, SE = 0.2, *p* < 0.000).

**Table 4:** Results of likelihood ratio test comparing models without a random factor QTYPE (model 1) and with a random factor QTYPE (model 2).

Reaction times (RTs)									
Model 1: RT ∼ ACT * WO * FREQ + (1| subj) + (1| item)									
Model 2: RT ∼ ACT * WO * FREQ + (1| subj) + (1| item) + (1| qtype)									

	**Df**	**AIC**	**BIC**	**logLik**	**Chisq**	**Chi**	**Df**	**Pr( > Chisq)**

Model 1	11	96613	96688	–48295				
Model 2	12	95986	96068	–47981	628.56		1	<0.0001

**Accuracy of responses (ANS)**								

Model 1: ANS ∼ ACT * WO * FREQ + (1| subj) + (1| item)								
Model 2: ANS ∼ ACT * WO * FREQ + (1| subj) + (1| item) + (1| qtype)								

	**Df**	**AIC**	**BIC**	**logLik**	**Chisq**	**Chi**	**Df**	**Pr( > Chisq)**

Model 1	10	2777.3	2845.2	–1378.7				
Model 2	11	2587.2	2662.0	–1282.6	192.06		1	<0.0001

*The test was performed for reaction times and accuracy of the responses to the comprehension task.*

#### ERP data

**FIGURE 3:**
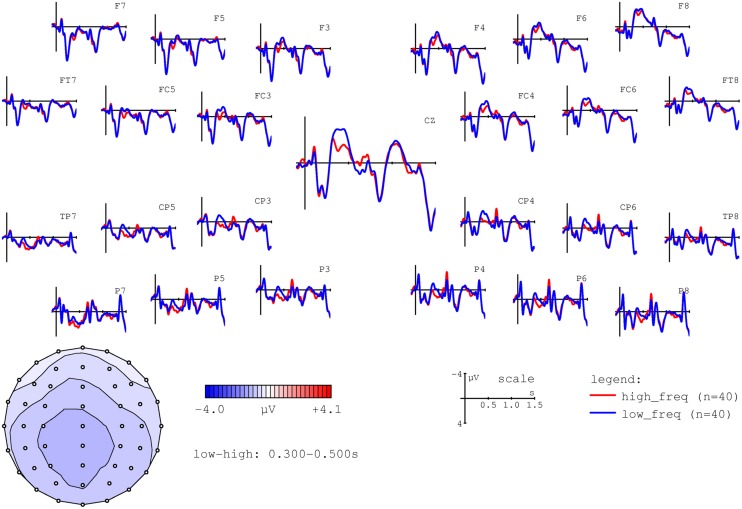
**Grand average ERPs time-locked to the second NP (onset at the vertical bar) for high (red line) and low frequency (blue line) nouns.** Negativity is plotted upward. The voltage map shows the topography of the effect (amplitudes for low – high frequency nouns in the 300–500 ms time window).

The main aim of this study was to test the hypothesis that individual nouns’ ACT values would correlate with modulations of the N400 at the position of NP2. Firstly, however, in order to ensure that our experiment replicated well-established ERP findings with respect to the N400, we performed a median split for lexical frequency at the position of NP2. As shown in **Figure 3**, this comparison indeed replicated the observation of higher N400 amplitudes for low versus high frequency words (e.g., Kutas and Federmeier, 2000).

**FIGURE 4:**
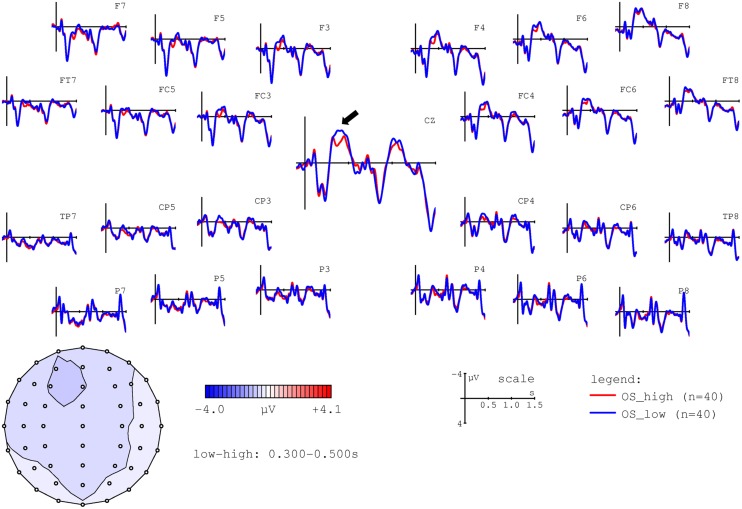
**Grand average ERPs time-locked to the second NP (onset at the vertical bar) for high (red line) and low ACT (blue line) nouns in object-initial sentences.** Negativity is plotted upward. The voltage map shows the topography of the effect (amplitudes for low – high ACT nouns in the 300–500 ms time window).

**FIGURE 5:**
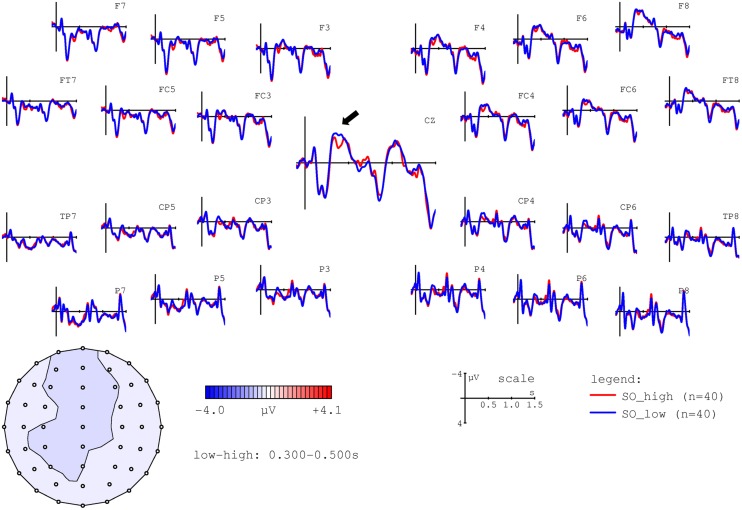
**Grand average ERPs time-locked to the second NP (onset at the vertical bar) for high (red line) and low ACT (blue line) nouns in subject-initial sentences.** Negativity is plotted upward. The voltage map shows the topography of the effect (amplitudes for low – high ACT nouns in the 300–500 ms time window).

In a second step, we examined the effects of noun-specific ACT values on ERPs at NP2. Recall that we predicted higher N400 amplitudes for nouns with low ACT values. Depending on the concrete hypothesis, this effect should be observable: (a) only in object-initial sentences, i.e., when the noun in question is the actor and, furthermore, predicted by the presence of an undergoer within the preceding sentence context (ACT as comparable to prominence scales); or (b) in both object- and subject-initial sentences (ACT as comparable to lexical properties such as concreteness). In order to visualize the effect of ACT, we thus performed a median split and contrasted high versus low ACT nouns in object-initial and subject-initial sentences. These comparisons are shown in **Figures 4 and 5**, respectively.

As is apparent from **Figures 4 and 5**, low ACT nouns engendered a more pronounced negativity between ∼300 and 500 ms post NP onset in comparison to their high ACT counterparts. This effect appears to be stronger in the object-initial sentences, i.e., when the critical NP was an actor rather than an undergoer.

**FIGURE 6:**
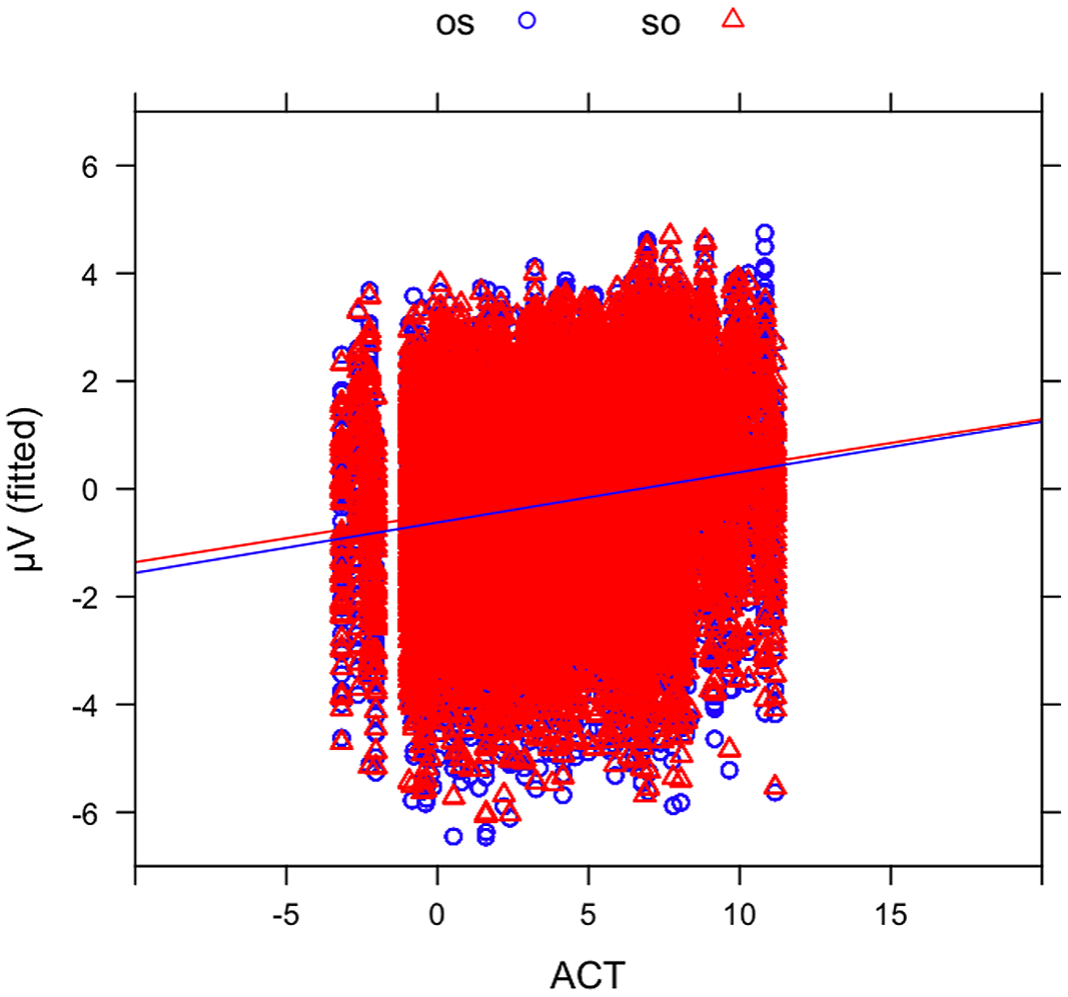
**Mean voltages in the N400 time window at the position of the critical noun phrase plotted against actorhood potential (ACT).** Sentences in object initial order (OS) are shown in blue circles and sentences in subject initial order (SO) in red triangles.

**FIGURE 7:**
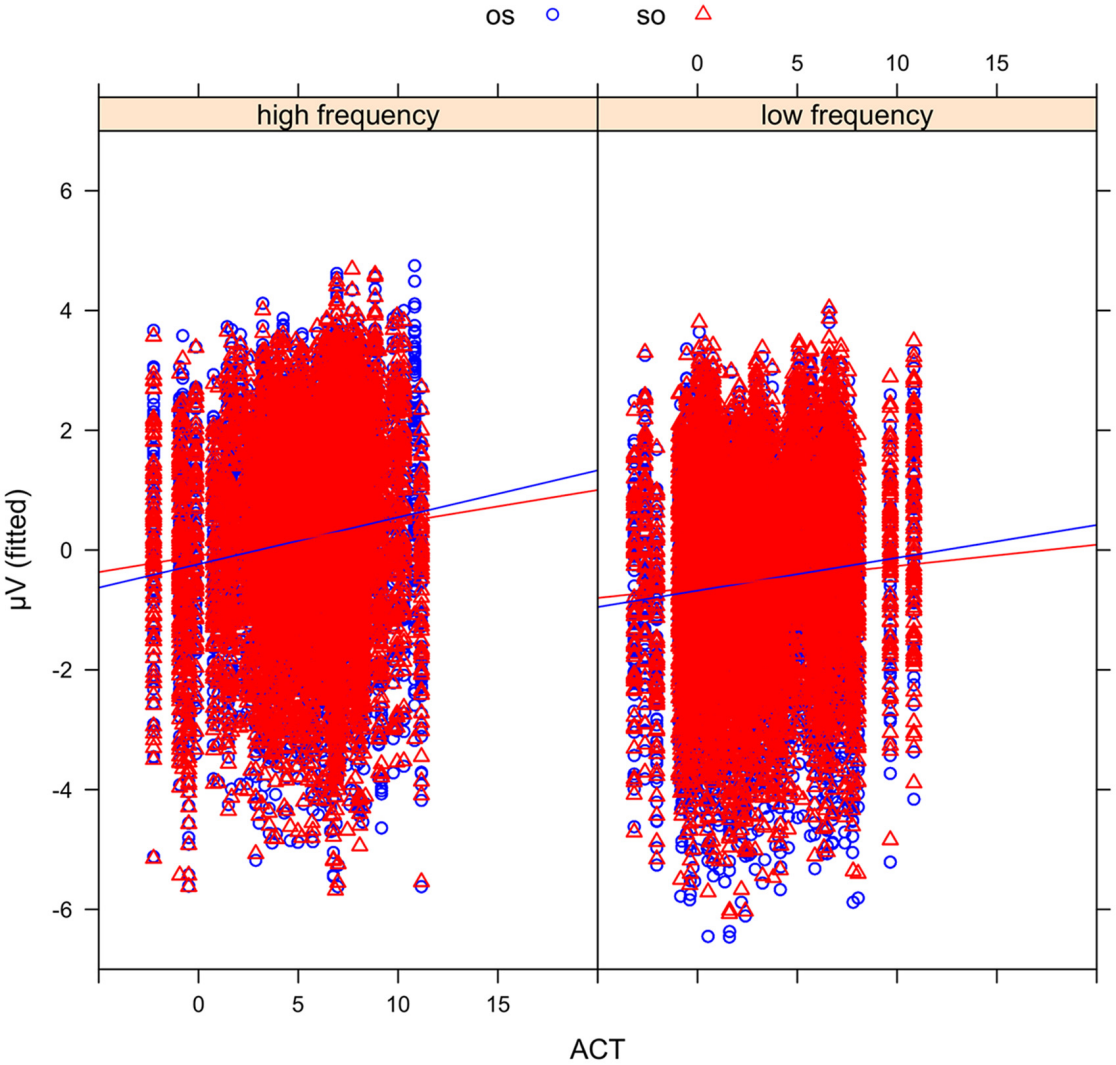
**Mean voltages in the N400 time window at the position of the critical noun phrase plotted against actorhood potential (ACT) for high frequency (FRQ; left panel) and low FRQ (right panel) nouns.** Sentences in object initial order (OS) are shown in blue circles and sentences in subject initial (SO) order in red triangles. Positive slopes indicate larger (i.e., more negative) N400 amplitudes for nouns with a low ACT value. Note that this figure also demonstrates the correspondence between the mixed model fits and the observed ERP pattern: the N400 amplitude difference for low versus high FRQ nouns (see **Figure 4**) is reflected here in the generally more negative amplitudes for low FRQ nouns **(right panel)** than their high FRQ counterparts **(left panel)**.

In order to examine the effect of ACT statistically, we fitted mixed-effects models including ACT as a fixed effect and ERP amplitude in the N400 time window (300–500 ms) as the dependent variable (see Materials and Methods). The best-fitting model (see the Supplementary Materials for a full specification), which provided a significantly better fit to the data than a comparable model without the factor ACT [likelihood ratio test: X^2^(6) = 781.9, *p* < 0.0001], showed an interaction of WO ^∗^ FRQ ^∗^ ACT (see **Figure 6**). This interaction was driven by more negative-going voltage changes in the OS condition (CE = –0.004, CI = –0.006 to –0.002, *p* < 0.000) with increasing ACT and FRQ values. Note that positive slopes in the figures indicate larger (i.e., more negative) N400 amplitudes for nouns with a low ACT value. **Figure 7** illustrates the source of the interaction by showing the effect of ACT in subject- and object-initial orders for high and low frequency nouns, respectively.

**FIGURE 8:**
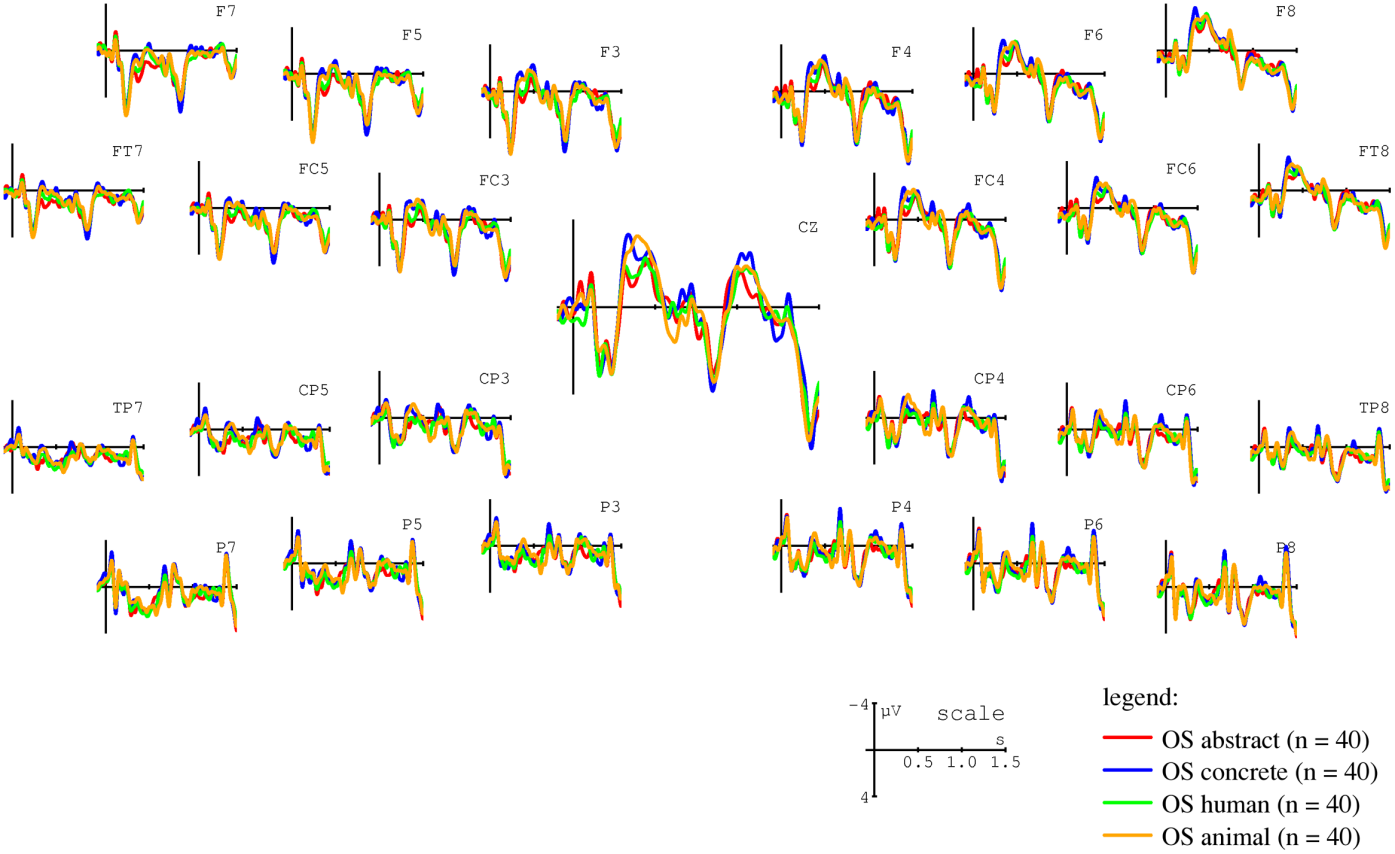
**Grand average ERPs time-locked to NP2 (onset at the vertical bar) in object-initial sentences.** The figure contrasts human (hum; green line), non-human animate (yellow line), concrete inanimate (con; blue line), and abstract inanimate (abs; red line) actor arguments. Negativity is plotted upward.

The results reported above provide compelling evidence for an ACT-based modulation of N400 amplitudes during sentence comprehension. In a final analysis step, we sought to examine how these effects relate to the animacy effects reported for actor arguments (following undergoers) in previous studies (e.g., Weckerly and Kutas, 1999; Roehm et al., 2004; Philipp et al., 2008). In particular, we wanted to ensure that the ACT-based effects reported above cannot be reduced to underlying animacy differences (i.e., to a correlation between ACT values and animacy/humanness). **Figure 8** shows ERPs for actor nouns in each of the animacy categories used in the present study.

As is apparent from **Figure 8**, the present study replicates several previously reported N400 modulations: an N400 effect for non-human versus human actors (e.g., Weckerly and Kutas, 1999; Roehm et al., 2004; Philipp et al., 2008) and for concrete versus abstract inanimate nouns (e.g., Kounios and Holcomb, 1994; West and Holcomb, 2000).

**FIGURE 9:**
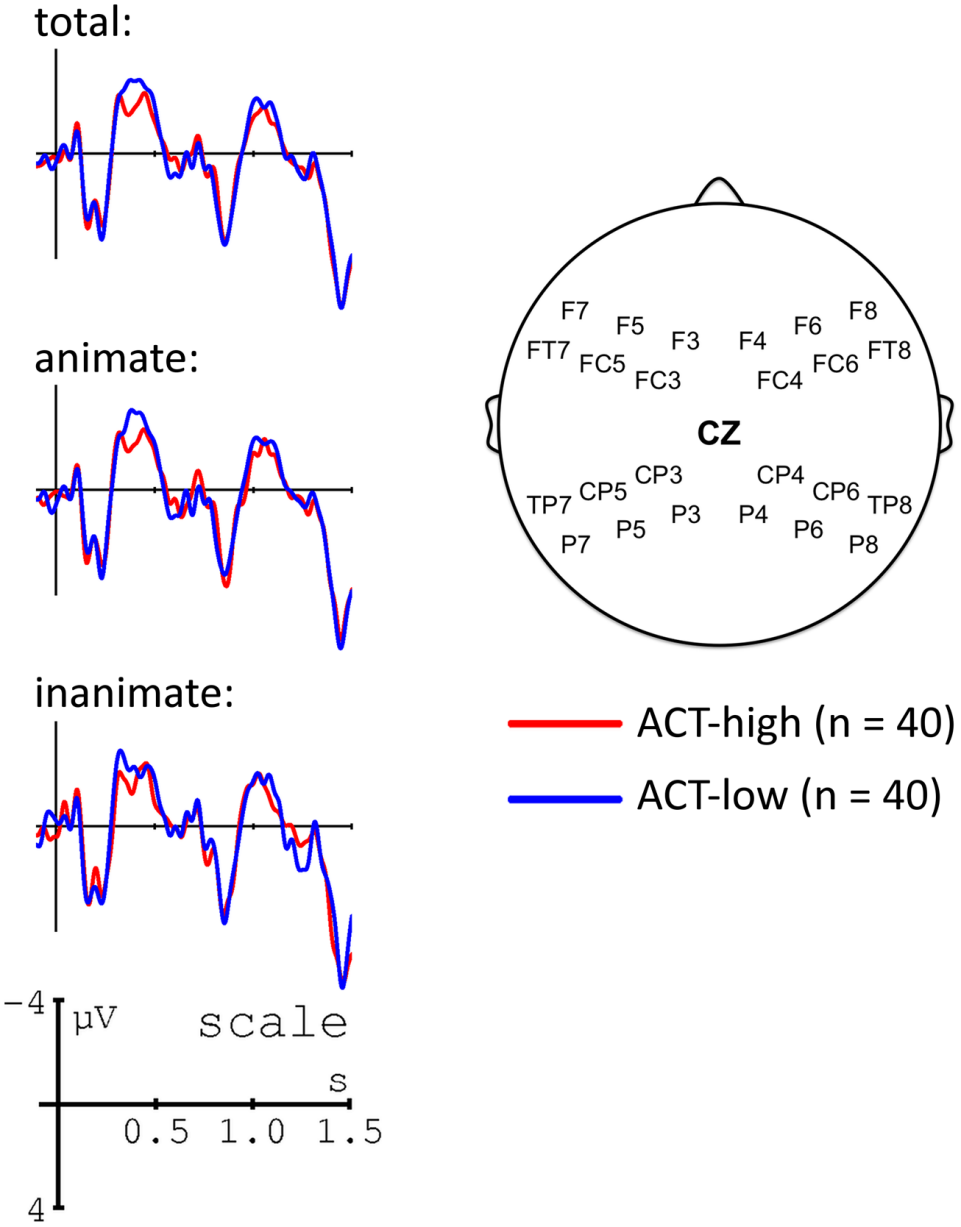
**Grand-average ERPs at the position of NP2 in object-initial sentences (onset at the vertical bar) at electrode FC3.** The figure contrasts high ACT (red trace) and low ACT (blue trace) nouns for animate and inanimate nouns, respectively. Negativity is plotted upward.

**FIGURE 10:**
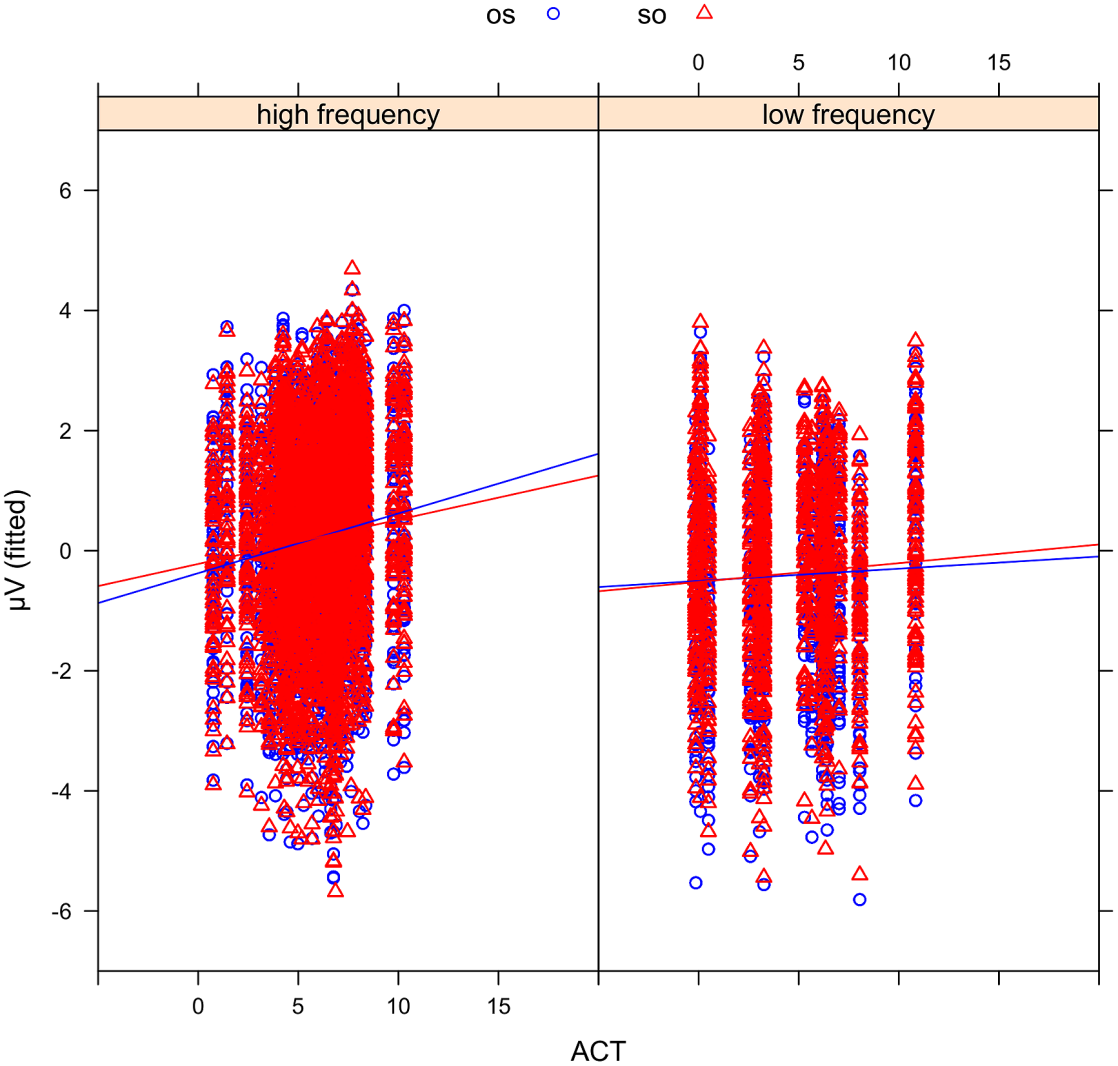
**Mean voltages plotted against actorhood potential (ACT) for high (left panel) and low frequency (right panel) human nouns only.** Sentences in object initial order (OS) are shown in blue circles and sentences in subject initial order (SO) in red triangles.

However, the animacy-based effects cannot account for the ACT effects reported above. As is apparent from **Figure 9**, an ACT-based N400 modulation was observable for both human and inanimate nouns. Furthermore, an additional mixed effects model analysis involving only human nouns replicated the effects reported for all nouns above. **Figure 10** shows the effects the ACT × FRQ interaction on ERP amplitude in this analysis (for further information regarding the best-fitting linear mixed effects model, see the Supplementary Materials).

### DISCUSSION

The ERP results from Experiment 2 provide strong converging support for the assumption that an individual noun’s inherent potential for actorhood (ACT value) influences online language processing. ACT values modulated N400 amplitude at the position of the critical noun and this modulation was stronger for object- than for subject-initial sentences, i.e., for critical nouns that were actors following an unambiguously marked undergoer. Interestingly, the effect was not confined to object-initial orders, but was also present in subject-initial orders, albeit in a weaker form. This pattern of results speaks in favor of a strong degree of lexicalization of the ACT value. The assumption of lexicalization is further supported by the observation that effects of ACT on the N400 were modulated by word frequency, with high frequency words showing stronger ACT effects than low frequency words.

## GENERAL DISCUSSION

We have presented two experiments on the inherent potential for actorhood of individual nouns and how it impacts upon sentence comprehension. In Experiment 1, a questionnaire study, we obtained values for a number of actorhood-related dimensions for German nouns. By means of a structural equation model, these were used to calculate an index of each noun’s potency to act (ACT). Experiment 2, an ERP study, demonstrated that a noun’s ACT value modulates the N400 component during sentence comprehension, a component which has been shown to be sensitive to the processing of actorhood in previous research. Nouns with a low ACT value engendered more negative-going N400 amplitudes and this correlation was more pronounced when the critical noun was designated as an actor by morphosyntactic cues and preceded by an unambiguously marked undergoer, and for high frequency as opposed to low frequency nouns. In the following, we begin by discussing the interaction between WO and ACT, before moving on to discuss the difference between actorhood potential and actor identification and the effects of lexical frequency. Here, we will argue that our results provide evidence for a lexicalized encoding of potency to act that is (at least partially) independent of prominence scales such as animacy. Finally, we turn to consequences for models of language processing.

### POTENCY TO ACT AND WORD ORDER

The results of Experiment 2 demonstrated a stronger ACT-based modulation of the N400 for object-initial sentences. In these structures, the critical noun was the actor of the sentence and, since it was preceded by a clearly case-marked undergoer (the accusative wh-pronoun *wen*), it was predicted by the processing system: an initial accusative undergoer requires a following nominative actor, but not vice versa, hence resulting the asymmetrical prediction (see e.g., Gibson, 1998, 2000; Bornkessel et al., 2004a; Schlesewsky and Bornkessel, 2004; Wolff et al., 2008). Thus, the ACT value had a similar effect to manipulations of animacy in previous studies, in which an inanimate actor following an undergoer also engendered a more pronounced N400 (e.g., Weckerly and Kutas, 1999; Roehm et al., 2004; Philipp et al., 2008). This finding therefore suggests that a noun’s potency to act is not only determined by prominence scales such as animacy, but also by fine-grained lexical-semantic features inherent to the individual noun itself. This conclusion is further supported by the observation of an ACT-based N400 modulation when only human nouns were considered (see **Figures 9 and 10** and the Supplementary Materials), thereby demonstrating that the effects observed here were not driven by the different animacy classes included in the stimulus materials.

These findings provide the first demonstration that an individual noun’s potency to act can be assessed fully independently of verb information. They thereby go beyond previous studies showing that verb-noun combinations can induce role typicality effects (e.g., McRae et al., 1997, 1998; Corrigan, 2001, 2002), thus further strengthening the notion of incremental thematic interpretation in verb-final sentences (Bornkessel et al., 2003; Kamide et al., 2003; Schlesewsky and Bornkessel, 2004; Bornkessel and Schlesewsky, 2006). As we have previously argued, verb-independent thematic interpretation presupposes the assumption of generalized semantic roles (Bornkessel et al., 2003). However, the assignment of nouns to these roles appears to go beyond the previously demonstrated influence of prominence scales such as animacy (Weckerly and Kutas, 1999; Roehm et al., 2004; Philipp et al., 2008) and case marking (Frisch and Schlesewsky, 2001, 2005) and to also rely on the experience-based ascription of properties which attest to an individual entity’s potency to act. Note that “act” here includes here the full range of roles associated with the generalized semantic role actor, i.e., not only the capacity for voluntary, goal-directed action, but also the ability to “bring about change” evident in inanimate causers (see **Figure 9** for an ACT-based modulation of N400 effects for human and inanimate nouns) and the capacity to experience mental states. To re-emphasize the independence of these results from animacy distinctions, recall that entities that can cause goal-directed action [see the discussion of Primus’s (2011) findings in Section “Identification of Suitable Rating Scales”] or that are associated with a high level of emotional arousal appear to be powerful sources of change and are treated as such by the language processing system. As shown by our second questionnaire study, the combination of these individual components in a noun’s overall ACT value not only predicts ERP responses but also correlates with native speakers’ intuitions about a noun’s capacity for actorhood.

However, the additional finding of a (smaller) ACT effect in subject-initial sentences suggests that goodness-of-fit to the actor role in the current sentence does not account for the complete set of results. Rather, the data suggest that, in addition to modulating actor fit, ACT behaves like a purely lexical feature (e.g., concreteness, frequency) in that it influences N400 amplitude for every noun independently of its thematic and syntactic role. This observation attests to the importance of the actor concept beyond the level of sentence processing: assuming that “any factor that facilitates lexical access should reduce N400 amplitude” (Lau et al., 2008, p. 921), our findings support the claim that high actorhood potential facilitates lexical access. We envisage this observation as resulting from the higher conceptual accessibility for nouns with a high actorhood potential -analogous to the higher conceptual accessibility that is often assumed for animate as opposed to inanimate entities (see Branigan et al., 2008, for discussion). Following Keil (1979), Bock and Warren (1985), and Branigan et al. (2008) describe conceptual accessibility in terms of the number of pathways to a concept, with more pathways correlating with easier lexical retrieval. Pathways, in turn, are defined in terms of “predictability” or the number of conceptual relations into which an entity can enter: “[A] human being is highly predicable because he or she can enter into many relations (e.g., growing, eating, sleeping, talking, ironing, and arguing). Spiders can enter into fewer relations (e.g., growing, eating and sleeping, but not talking, ironing, or arguing), and clouds still fewer.” (Branigan et al., 2008, p.174). This notion of predictability is, in fact, quite close to our notion of “potency to act,” as entities with a higher ACT value can be assumed to enter into a higher number of relations as the actor of an event. We shall return to the broader consequences of positing higher lexical accessibility for high ACT nouns below.

To summarize, the ERP results from Experiment 2 suggest that the ACT value of individual nouns impacts upon online sentence processing in two ways: (a) by reflecting the goodness-of-fit to a predicted actor role in the current sentence even before the verb is reached (as shown by the stronger ACT effect for actors as opposed to undergoers); and (b) by modulating lexical access (as shown by the presence of an ACT-based N400 modulation for undergoers as well as actors).

### ACTORHOOD VERSUS ACTOR POTENTIAL

In the preceding discussion, we have suggested that a noun’s ACT value influences online sentence comprehension in two different ways, namely by influencing actor identification within the current sentence and by modulating lexical access. As this is clearly a claim with potentially far-reaching implications, this subsection examines additional evidence in its favor.

Firstly, why should these two dimensions be distinct? Clearly, it is important to separate an entity’s inherent “potency to act,” which is independent of the current sentence context, from the status of a sentence participant as an actor or non-actor within a particular sentence context, which is independent of that referent’s inherent potency to act. Consider, for example, the two nouns *Ärztin* (“female doctor,” ACT value = 8.0) and *Greisin* (“very old woman,” ACT value = –0.14), which differ clearly with regard to their inherent potency to act. Nevertheless, a sentence in which the low ACT noun acts upon the high ACT noun is perfectly possible, e.g., *Die Greisin schubste die Ärztin die Treppe hinunter* (“The old woman pushed the doctor down the stairs.”). Thus, in addition to being able to determine inherent (lexical) actor potential, the language processing system must have the ability to infer which argument is the actor within the current sentence context (actor identification).

We have argued extensively in previous publications that actor identification is accomplished with reference to linguistic prominence scales such as those in (7) (for detailed discussion, see Bornkessel-Schlesewsky and Schlesewsky, 2009):

(7) Linguistic prominence features related to actor identification_
    (a) +animate/+human (follows from control/volition and sentience)
    (b) +definite/+specific (follows from independent existence; see Primus, 1999)
    (c) +1st position (correlates with actorhood cross-linguistically; see Tomlin, 1986)
    (d) +nominative (correlates with actorhood in nominative-accusative languages)

While the prominence feature animacy/humanness (a) in part also defines the ACT value (see Experiment 1), properties (b) through (d) are lexically independent. They thus ensure that nouns which do not have a high degree of inherent actorhood potential can nevertheless be realized as actors. The degree to which the prominence features in (7) determine actor identification within a particular language depends on their relative weighting within the language in question (cf. Bates and MacWhinney’s Competition Model, e.g., MacWhinney and Bates, 1989). Thus, while position (or WO; property c) is particularly important in English, case marking (property d) is particularly central in German (MacWhinney et al., 1984). In other languages, by contrast, animacy plays a comparable role: in the languages Fore and Awtuw (spoken in Papua New Guinea), for example, sentences are interpreted according the animacy hierarchy (human > non-human animate > inanimate) such that, in the absence of additional marking, a sentence with a non-human and a human argument will always be interpreted with the human as actor (Scott, 1978; Feldman, 1986; for discussion within a psycholinguistic context, see Bornkessel-Schlesewsky and Schlesewsky, 2009). Animacy thus has a very different status to ACT here, since it determines interpretation independently of a noun’s inherent actorhood potential. Importantly, even though prominence scales can clearly override ACT in actor identification within a particular sentence context, we nevertheless assume that ACT influences a noun’s goodness-of-fit to the actor role in a given sentence (i.e., a low-ACT noun is a “worse” actor candidate than a high-ACT noun; see Consequences for Models of Language Processing below for an interpretation in terms of constraint-based sentence processing).

We thus propose that there are two dimensions to actor processing during online sentence comprehension: a noun’s lexicalized potency to act (ACT), which influences lexical access (in the sense of conceptual accessibility/ease of retrieval as discussed in Section “Potency to Act and Word Order”), and actor identification as determined via prominence scales and modulated via ACT values. Indeed, a dissociation along these lines even appears to be neurobiologically plausible: we have recently proposed that the two major functional-neuroanatomical processing streams for speech and language in the human brain (the antero-ventral and postero-dorsal streams; cf. Rauschecker and Scott, 2009) are responsible for the recognition and sequence-independent (commutative) combination of auditory objects (e.g., phonemes, morphemes, words, phrases; see also DeWitt and Rauschecker, 2012) and the processing of linguistic sequences, respectively (Bornkessel-Schlesewsky and Schlesewsky, 2013a; Bornkessel-Schlesewsky et al., in press). Lexicalised potency to act, as a property of individual referents, clearly falls into the former domain and thereby accords with the overall function of the antero-ventral stream in sentence processing. Actor identification, by contrast, is dependent at least in part on the sequence in which the words in a sentence are encountered and thereby falls into the domain of the postero-dorsal stream (see Consequences for Models of Language Processing). Animacy, as one of the dominant features related to actorhood across languages, thereby plays a twofold role in that it is both a determinant of ACT and a lexically independent prominence scale.

But why should the ACT effect be larger for object-initial than for subject-initial sentences in the present study (i.e., for actor as opposed to undergoer arguments)? We assume that this reflects both actor identification and lexical access. On the one hand, a low-ACT noun leads to a mismatch with a predicted, prototypical actor (reflected in the N400 effect), thus modulating actor identification. On the other hand, we posit that the increased N400 for low-ACT actor nouns is due to a top–down modulation of lexical access: since the processing system has already encountered an unambiguously marked undergoer, it expects to process an actor, thus leading to a stronger preactivation of good actor candidates. When a noun with a low ACT value is subsequently encountered, an N400 effect results and this effect is more pronounced than in the absence of actor preactivation (as in a subject-initial sentence when no prediction for an upcoming actor is required).

### EFFECTS OF LEXICAL FREQUENCY

In view of the proposal that ACT is a lexically encoded feature, the additional observation of an interaction between ACT and frequency is not surprising. Recall that the correlation between a noun’s ACT value and N400 amplitude was modulated by frequency such that it was stronger for high frequency as opposed to low frequency nouns. While this interaction was not predicted prior to the experiment, it fits very well with the perspective on an entity’s lexicalized potency to act that was advocated in the previous sections. High frequency nouns are those with which people have a lot of experience. Hence, they will have more detailed and precise knowledge about the properties of these nouns (as well as the entities that they denote) and the actions that they are likely to perform. This does not mean that actor potential cannot be calculated for low frequency nouns – Experiment 1 demonstrates that it can. However, the semantic properties relevant to determining actor potential may not be as readily, and as rapidly, available for low frequency nouns as for their high frequency counterparts. In other words, something akin to an ACT value may be “precompiled” and lexically encoded for high but not for low frequency nouns. This claim is reminiscent of the proposal that highly frequent words may be stored as full forms in the lexicon even if they are regular (e.g., Laine et al., 1995; Baayen et al., 1997; Alegre and Gordon, 1999). Here, we suggest that high frequency of occurrence may lead to the lexical storage of additional properties such as actor potential.

### CONSEQUENCES FOR MODELS OF LANGUAGE PROCESSING

The discussion in the previous section revealed that the present findings call for two separate dimensions of actorhood computation: an experience-based, lexically encoded representation of actorhood potential, and a prominence-based, computational mechanism for calculating goodness-of-fit to the actor role in a particular sentence context. In the following, we discuss the consequences of this observation for theories of sentence processing in general and, more specifically, for neurocognitive theories of language comprehension.

Firstly, our overall result can be couched within constraint-satisfaction architectures of language comprehension (cf. McRae and Matsuki, 2013, for an overview), assuming that (a) constraint-satisfaction applies incrementally and, if necessary, independently of verb-based representations; (b) actor identification is governed by constraints determining goodness-of-fit to the actor role in a given sentence; and (c) the impact of lexical potency to act (ACT) is determined by constraints at least partially separable from those involved in actor identification. As discussed in detail above, actor identification constraints must be able to override ACT-based constraints, since low-ACT nouns could not otherwise be interpreted as actors. This suggests that the two types of constraints are at least quantitatively distinct. On the basis of our results (i.e., the differential N400 modulations observed for ACT-values as opposed to animacy as an actor-identifying prominence feature), we would assume that they are, in addition, qualitatively distinct. This proposal leads to the testable prediction that ACT-values should be independent of (or at least not determined by) the relative weighting of the animacy prominence feature in a given language (i.e., we would still expect animacy to play a role in determining ACT-values in a language such as English, even though it is a relatively weak cue to overall sentence interpretation in this language; MacWhinney et al., 1984).

Turning now to neurocognitive models of language processing, a central consequence of our results for such models is that they need to be able to incorporate the two independent dimensions of actor processing identified here. Indeed, all established neurocognitive models of sentence processing provide a principled means of dissociating lexicalized information from computational factors. In Hagoort’s (2003, 2005, 2013) Memory, Unification and Control (MUC) model, this is achieved via the distinction between unification (offering the potential to derive prominence computation) and memory (offering the potential to derive retrieval of lexicalized actorhood potential). Like the MUC approach, Ullman’s (2001, 2004) declarative/procedural model of language processing also distinguishes combinatory mechanisms, which form part of procedural memory, from lexical information, which forms part of declarative memory. Friederici’s (2002, 2012) neurocognitive model likewise assumes a principled separation between the processing of morphosyntactic and lexical-semantic information, which are processed independently but in parallel in the model’s second phase. Finally, Tyler and Marslen-Wilson (2008) put forward a neurocognitive model of language processing focused on “three aspects of language function” and posit that “two of these, involving inflectional morphological and syntactic processes, clearly group together in distinction from the third, semantic function” (Tyler and Marslen-Wilson, 2008, p. 1051). They thereby once again echo the purported distinction between combinatory and lexical/conceptual aspects of language. Thus, all of these approaches could, in principle, easily account for lexically based effects of actorhood by simply introducing a word-specific actor potential value into each lexical entry of a potentially “nouny” word.

By contrast, combinatory effects based on prominence information such as animacy are not naturally accounted for by any of these models. Both Friederici’s and Ullman’s models subscribe to a traditional (categorical) notion of linguistic rules. It is therefore not clear how weighted, combinatorial interactions – as required in prominence-based actor identification – might be derived. In addition, these approaches – like Tyler and Marslen-Wilson’s account – assume a principled separation between syntax and semantics, thus rendering a direct interaction of semantic and morphosyntactic cues for actor identification problematic (see Bornkessel-Schlesewsky and Schlesewsky, 2009, for a general discussion of this issue). The MUC model is lexicalist in nature and thereby does not offer a natural way of dealing with pre-verbal argument interpretation in verb-final sentences (see Vosse and Kempen, 2000, for discussion of this topic in reference to the computational model of sentence processing on which the MUC model is based; and Bornkessel and Schlesewsky, 2006). In addition, the syntactic representations currently assumed (lexical frames) do not incorporate prominence features; thus prominence-based unification would appear to have to rely on an interaction with the lexicon (e.g., in the sense of features such as animacy modulating competition for positions within a syntactic frame).

In contrast to the models discussed so far, our own neurocognitive model of language processing, the (extended) Argument Dependency Model [(e)ADM; Bornkessel, 2002; Schlesewsky and Bornkessel, 2004; Bornkessel and Schlesewsky, 2006; Bornkessel-Schlesewsky and Schlesewsky, 2008, 2009, 2013a] was developed precisely to account for computational (prominence-based) mechanisms of actor identification, since these appear to constitute a cross-linguistically stable aspect of the language processing architecture. Thus, one of the model’s strengths has traditionally lain in its ability to derive the relational – rather than the lexical – aspects of actor processing. The most recent version of the eADM (Bornkessel-Schlesewsky and Schlesewsky, 2013a; Bornkessel-Schlesewsky et al., in press), however, provides a principled, neurobiologically grounded motivation for the distinction between lexical and computational facets of actorhood.

In its latest version, the eADM posits a fundamental, functional-neuroanatomical computational division of labor between the antero-ventral and postero-dorsal processing streams. As already noted in Section “Actorhood Versus Actor Potential,” the model assumes that the dorsal stream engages in sequence processing (non-commutative combinatorics), while the ventral stream engages in commutative combinatorics (i.e., the order-independent combination of features to form successively more complex feature or dependency structures). Processing in both streams is organized in a hierarchical manner in accordance with the neurobiological principle of hierarchical processing (e.g., Felleman and Van Essen, 1991; Rauschecker, 1998; Rauschecker and Scott, 2009; DeWitt and Rauschecker, 2012) and classic assumptions regarding the structure of complex cognitive models (Simon, 1962; Newell, 1990). This means that, as information flows along the streams, the representations that are processed are assumed to become increasingly complex.

Crucially, the representations identified and processed by the antero-ventral stream are assumed to correspond to “auditory objects” of successively increasing size – a proposal which was originally put forward on the basis of single unit recordings in non-human primates (Rauschecker, 1998; Rauschecker and Scott, 2009). In the language domain, the smallest of these auditory objects might correspond to the phoneme and the largest to complex phrases or perhaps even sentences (DeWitt and Rauschecker, 2012), and the eADM posits that word-level representations can be modeled via “actor-event schemata” (AE-schemata). These schemata are lexical objects which are unified with one another to form larger interpretive units (for details, see Bornkessel-Schlesewsky and Schlesewsky, 2013a). They are associated with an experience-based value of actor suitability, which comes into play whenever a given schema is identified as corresponding to a “nouny” constituent by the current sentence context. AE-schemata thus provide a natural means of incorporating word-based actor potency information which is, moreover, neurobiologically motivated by the more general function of the antero-ventral stream in the identification of successively complex auditory objects.

The postero-dorsal stream, by contrast, engages in the predictive processing of information for which sequential order is important. This includes prosodic segmentation and basic syntactic analysis (in the sense of syntactic processing entailing the analysis of a sequence of linguistic categories) as well as the identification of the actor for a given sentence (event). Actor identification in this sense must clearly be performed by the postero-dorsal as opposed to the antero-ventral stream since it presupposes processing of the linguistic input in time – i.e., the position in which an argument appears within the category sequence constituting a sentence is clearly relevant to actor identification and this is the case in all languages, even though some may weigh this information more strongly than others. In this way, the lexically independent aspect of actor computation is also neurobiologically grounded within the latest version of the eADM.

In addition to the consequences for neurocognitive models of language processing discussed above, the present findings also have implications for models of the N400 component. Traditionally, prominent approaches to the N400 either stressed the role of lexical preactivation (“lexically based” approaches) or of integration into the current sentence context (“integration-based” approaches) in deriving N400 amplitude modulations (see Lau et al., 2008, for a review of both types of models). According to lexically based accounts (e.g., Kutas and Federmeier, 2000; Lau et al., 2008; Brouwer et al., 2012), N400 amplitude is reduced when an element is preactivated in semantic memory. In accordance with this view, it has been suggested that the language processing system establishes predictions about upcoming elements which, in constraining contexts, can be specific enough to lead to the anticipation of individual words (e.g., Wicha et al., 2004; DeLong et al., 2005; Van Berkum et al., 2005; Federmeier, 2007; Otten et al., 2007). The integration-based approach (e.g., Hagoort, 2008), by contrast, posits that N400 amplitude reflects the ease with which a word can be integrated into the current sentence and discourse context (though note that this does not necessarily presuppose that lexical access must have been completed in order for integration to begin). Notably, however, these two perspectives (preactivation due to top–down predictability versus bottom–up integrability) are not mutually exclusive. For example, Federmeier (2007) proposed an account of the N400 involving both information sources: top–down, predictive mechanisms (based on language production) operate primarily in the left hemisphere; the right hemisphere, by contrast, is assumed to process information in a more strongly feed-forward (bottom–up manner), thus ensuring that important stimulus-based information is not missed as a result of too strong predictive influences. More recently, Baggio and Hagoort (2011) offered a somewhat different suggestion regarding the integration of top–down and bottom–up information sources in the N400. In their view, top–down influences reflect unification requirements – processed in inferior frontal cortex – while bottom–up influences reflect the spreading of activation within semantic memory in temporal cortex. Finally, Lotze et al. (2011) demonstrated that bottom–up information need not be lexical to exert a profound effect on the N400, observing that the effects of predictability/integration can be almost entirely neutralized by paralinguistic orthographic cues (capitalization). Their “bidirectional coding account” thus stresses not only the integration of top–down and bottom–up information sources but also the degree of match between them.

The present results further support the idea that bottom–up/top–down integration is a crucial explanatory concept with regard to the N400. The fact that the lexical ACT effect was observed for all arguments (i.e., not just those that were actors in the current sentence context), calls for a purely bottom–up explanation. Nevertheless, as argued above, this effect can be amplified by top–down information, when prediction for an upcoming actor leads to lexical preactivation of good actor candidates. The prominence-based computation of actorhood, by contrast, generally relies on the integration between top–down (e.g., WO, case marking of previous arguments) and bottom–up (e.g., case marking and animacy of the current argument) information sources. This suggests that the overall N400 response could comprised a family of N400 effects, displaying differential sensitivity to the balance between top–down and bottom–up information sources (see Haupt et al., 2008, for the assumption of an N400 family). Furthermore, our findings indicate that predictability-based top–down influences on the N400 are not confined to the anticipation of individual words, but rather allow for the prediction of more abstract notions such as a generalized participant role (actor). In other words, predictability here is not driven by the build-up of a highly specific sentence context, but rather by more general cues regarding the nature of upcoming classes of participants.

To summarize, while most established neurocognitive models of sentence processing do not predict the lexicalization of actorhood, it appears that this observation could be incorporated into all approaches in a relatively straightforward manner. The distinction between lexicalized and prominence-based actorhood, by contrast, appears more difficult to integrate into most existing models with the exception of the eADM. With regard to models of the N400, the present findings support the notion of top–down/bottom–up integration, while additionally emphasizing the ability of the language comprehension system to predict not only individual words but also more general stimulus categories (cf. also Szewczyk and Schriefers, 2013).

## CONCLUSION

We have presented a detailed investigation of how linguistically encoded actors – participants responsible for the event being described – are processed during language comprehension. On the basis of a questionnaire study and an associated structural equation model as well as an ERP study, we have argued that an individual’s potency to act is encoded at the lexeme level for individual words and that this information is thereby considerably more fine-grained than previous investigations with macroscopic noun classes based on prominence scales (e.g., animacy) have suggested. Since the effect of the lexeme-specific actor potential is modulated by word frequency, we further propose that it is conditioned by the hearer/speaker’s experience with a particular lexeme and, accordingly, the types of actions that the entity denoted by that lexeme is likely to undertake. We have argued that the present findings are indicative of a twofold influence of actorhood on language comprehension: (a) via a lexicalization of actorhood potential that is based on one’s experience about the suitability of individual nouns to fill the actor role and that modulates lexical access whenever a “nouny” constituent is encountered; (b) competition for the actor role within a given sentence context, in which a noun’s inherent suitability as an actor interacts with other information sources (e.g., WO, case marking). Thus, the effects of actorhood observed result from the interplay of bottom–up (lexical) and top–down (context-based expectation for an actor) information sources. We suggest that the special status of the actor role may follow from domain-general properties of human cognition, particularly the independently required need to recognize the initiators of actions – and their intentions – in the world around us.

## Conflict of Interest Statement

The authors declare that the research was conducted in the absence of any commercial or financial relationships that could be construed as a potential conflict of interest.

## Footnotes

1 http://www.limesurvey.org

2 http://wortschatz.uni-leipzig.de
