# Supplementary material for: Two routes to actorhood: lexicalized potency to act and identification of the actor role
Source: Front Psychol. 2015 Jan 30;6:1. doi: 10.3389/fpsyg.2015.00001 (PMC4311632; doi:10.3389/fpsyg.2015.00001)

Supplementary Material A: List of nouns used in Experiment 1 and their factor values

Table A1 shows the nouns used in Experiment 1 together with their English translations, their mean ratings on the 9 scales that entered the best-fitting structural equation model and their factor values.

Table A2 shows separate ratings and factor values for the different noun types used (humans, animals, concrete objects, abstract concepts).

Table A3 shows mean actor ratings and standard deviations of validation test for the different noun types used (humans, animals, concrete objects, abstract concepts).

Table A1: Factor values per noun

noun	translation	cond	logfreq	VAL2	PLE2	APP2	POW	STR	SIZ	ANI	CON	AIM	EVA	POT	HUM	ACT
Adler	eagle	B	7.51	1.68	1.57	2.46	2.36	2.36	1.71	2.79	1.75	2.04	3.72	3.37	1.59	9.17
Amme	wet nurse	A	3.61	2.00	1.42	0.91	0.97	1.06	0.24	1.79	2.03	1.88	2.91	1.20	1.62	6.19
Anstand	decency	D	6.14	2.26	2.12	1.47	0.94	0.94	0.79	-0.68	0.76	1.65	4.17	1.08	0.22	5.87
Anwalt	lawer	A	9.02	1.15	1.03	0.44	2.06	1.47	0.59	1.88	2.38	2.62	1.57	2.32	1.91	6.43
Ärger	trouble	D	8.49	2.09	2.34	1.94	1.03	0.66	0.78	0.94	0.56	0.94	4.55	0.88	0.36	6.01
Ärztin	physician	A	6.87	2.18	1.62	1.06	1.74	1.24	0.32	2.26	2.62	2.59	3.34	1.88	2.16	8.00
Autor	author	A	8.83	1.71	1.54	0.79	1.71	1.07	0.46	2.14	2.64	2.07	2.74	1.72	2.15	7.11
Becher	mug	C	6.05	1.47	1.19	0.84	-1.31	-0.56	-0.50	-2.38	-2.44	0.50	2.22	-1.86	-2.52	-2.04
Betrug	fraud	D	8.10	2.61	2.55	2.30	1.45	1.18	0.91	-0.21	0.24	2.18	5.37	1.65	-0.09	7.45
Bettler	beggar	A	5.27	1.21	1.65	1.44	-2.06	-2.03	-0.62	1.09	1.65	-1.00	2.84	-3.72	1.21	0.09
Biber	beaver	B	5.32	1.03	1.06	1.12	0.03	1.44	-1.03	2.38	1.50	1.79	1.90	0.89	1.33	4.55
Bleistift	pencil	C	5.52	1.71	1.41	0.91	0.29	-0.21	-1.32	-2.21	-2.32	1.38	2.67	-0.46	-2.40	0.15
Bombe	bomb	C	8.33	2.74	2.76	2.38	2.59	2.71	1.62	-2.00	-1.74	1.74	5.74	3.84	-1.92	8.08
Büffel	buffalo	B	4.41	0.97	0.91	1.25	1.81	2.44	2.59	2.66	1.28	0.97	1.77	3.09	1.21	6.30
Buntspecht	gr. sp. woodpacker	B	2.77	1.19	1.50	1.66	-0.81	-0.56	-2.03	2.56	1.28	1.00	2.80	-1.55	1.20	2.68
Bussard	buzzard	B	4.49	1.64	1.39	1.86	1.75	1.93	0.82	2.68	1.68	2.32	3.15	2.54	1.52	7.77
Dackel	dachs hund	B	5.16	0.73	0.94	1.18	-0.85	-0.82	-2.12	2.12	1.30	-0.61	1.58	-1.81	1.13	0.76
Dame	lady	A	8.00	1.53	1.76	1.88	0.62	-0.03	0.15	2.38	2.32	1.26	3.47	-0.04	1.95	5.69
Decke	blanket	C	7.64	2.30	2.36	1.73	-0.09	0.30	1.64	-1.61	-2.15	-0.64	4.61	-0.19	-2.16	2.10
Delphin	dolphin	B	4.61	1.84	2.00	2.16	0.44	1.13	1.16	2.88	2.25	1.47	4.12	0.92	1.99	7.38
Dichter	poet	A	7.64	1.89	1.82	1.18	0.79	-0.07	0.00	2.14	2.75	1.18	3.40	0.03	2.24	5.95
Diebin	thief	A	3.61	1.84	1.97	0.89	0.58	0.42	-0.74	2.34	1.63	2.32	3.36	0.33	1.42	5.66
Diener	buttler	A	6.19	1.53	1.65	0.62	-1.06	-0.56	-0.12	1.97	2.21	2.15	2.62	-1.68	1.79	3.25
Doktor	doctor	A	6.85	1.91	1.28	0.47	1.59	0.78	0.44	1.91	2.50	2.22	2.44	1.37	2.00	6.34
Dose	can	C	6.16	1.18	0.79	1.04	-1.32	-0.68	-1.39	-2.71	-2.57	-0.75	1.68	-1.99	-2.68	-3.17
Ehre	honor	D	8.21	2.00	1.86	1.75	2.18	2.07	1.71	0.75	0.29	1.14	3.86	2.98	0.11	7.22
Ehrgeiz	ambition	D	7.31	1.41	1.00	0.79	1.97	1.50	1.53	0.47	0.21	2.59	1.95	2.31	0.00	4.88
Eifer	eagerness	D	6.23	1.52	1.27	0.85	1.82	1.82	1.30	1.24	0.39	2.30	2.35	2.50	0.28	5.68
Ekel	disgust	D	5.63	2.24	2.85	2.48	1.03	1.55	0.70	0.70	-0.06	0.61	5.51	1.70	-0.16	7.19
Ente	duck	B	6.55	1.12	1.18	1.21	-1.33	-1.12	-1.21	2.21	1.18	-0.18	2.16	-2.40	1.06	0.77
Erfolg	sucess	D	10.39	2.18	2.50	1.95	2.24	2.18	1.79	0.97	0.47	2.66	4.79	3.13	0.30	8.85
Eule	owl	B	4.53	1.47	1.38	1.44	0.50	0.38	0.06	2.09	1.81	1.13	2.76	0.25	1.51	4.79
Falter	moth	B	5.39	1.24	1.38	1.76	-1.35	-1.53	-1.79	2.56	0.79	-0.09	2.78	-2.80	0.82	0.78
Faultier	sloth	B	3.18	0.81	0.78	1.19	-0.81	-0.44	-0.06	1.44	0.97	-0.25	1.48	-1.40	0.76	0.78
Ferkel	piglet	B	4.90	1.18	0.82	1.03	-1.15	-0.42	-1.67	2.79	1.06	-0.21	1.72	-1.65	1.07	1.09
Fleischer	butcher	A	6.01	1.21	1.30	0.88	1.06	1.91	1.33	2.24	2.15	1.67	2.16	2.06	1.80	6.42
Fliege	fly	B	6.16	1.00	1.91	1.67	-1.73	-1.82	-2.61	2.64	-0.33	-1.12	3.09	-3.34	-0.02	-0.54
Flunder	flatfish	B	3.78	0.70	0.76	1.48	-1.55	-1.00	-1.03	1.30	0.45	-0.64	1.54	-2.43	0.34	-0.70
Forscher	scientist	A	9.49	2.24	1.36	0.58	1.70	1.03	0.73	1.70	2.61	2.58	2.84	1.68	2.05	7.18
Fortschritt	progress	D	7.81	1.88	1.62	1.15	2.35	1.94	1.85	0.82	0.06	2.38	3.16	2.98	-0.05	6.67
Freude	happyness	D	9.10	2.76	2.79	2.76	1.71	1.92	1.84	2.39	1.47	0.42	6.01	2.53	1.31	9.95
Freundin	friend	A	8.84	2.68	2.82	2.36	1.57	1.36	0.36	2.68	2.68	1.61	5.74	1.88	2.28	10.29
Frieden	peace	D	8.76	2.91	2.70	2.70	0.88	0.97	1.55	1.03	0.79	1.33	5.99	1.08	0.54	7.93
Friseur	hairdresser	A	6.21	1.45	1.61	1.03	0.64	0.24	-0.06	2.36	2.24	1.79	2.75	0.21	1.89	5.29
Fruchtsaft	fruit juice	C	3.83	2.21	2.04	1.07	-0.54	0.43	-0.11	-0.07	-1.93	-0.36	3.81	-0.41	-1.72	1.60
Fußball	football	C	9.78	1.44	1.18	0.97	0.68	1.24	0.50	0.94	-0.32	1.79	2.26	1.17	-0.32	3.54
Gärtner	gardener	A	6.83	1.61	1.39	0.45	-0.47	0.97	0.32	1.89	1.89	1.76	2.31	0.14	1.54	4.41
Gefahr	danger	D	9.80	2.50	2.71	1.57	1.93	1.64	1.50	0.68	-0.82	0.64	5.04	2.41	-0.74	6.86
Gefühl	feeling	D	9.24	1.91	1.36	1.67	2.12	1.97	1.88	2.27	0.73	0.58	3.22	2.85	0.72	6.93
Geiger	violinist	A	6.71	1.93	2.14	1.43	0.50	0.14	0.43	2.50	2.68	1.75	3.91	0.04	2.25	6.62
Goldfisch	goldfish	B	3.99	1.21	1.55	1.64	-1.55	-1.64	-2.06	2.18	-0.12	-1.00	2.86	-3.04	0.06	-0.36
Greisin	old woman	A	3.71	1.29	1.04	1.25	-1.25	-2.11	-1.79	0.82	1.61	-0.64	2.15	-3.26	1.13	-0.14
Grille	cricket	B	3.00	0.72	1.06	0.91	-0.75	-1.31	-2.25	2.06	0.56	-0.50	1.54	-2.20	0.56	-0.22
Hammer	hammer	C	7.39	1.06	1.28	0.97	1.50	2.28	0.63	-2.00	-2.22	1.63	2.08	2.69	-2.29	2.87
Hamster	hamster	B	4.98	1.21	1.58	1.42	-1.97	-1.95	-2.50	2.26	0.66	-0.82	2.76	-3.62	0.67	-0.39
Hausschwein	domestic pig	B	3.04	1.32	0.93	1.14	-0.89	0.75	-0.29	2.64	1.25	-1.00	2.01	-0.36	1.19	2.59
Heldin	heroine	A	5.89	2.47	2.00	2.21	2.24	2.38	1.74	2.62	2.68	2.65	4.63	3.31	2.27	10.84
Hoffnung	hope	D	8.10	2.50	2.28	2.22	1.91	2.09	1.47	1.16	1.06	2.03	4.96	2.81	0.78	9.03
Holztisch	wooden table	C	4.30	1.65	1.82	1.62	-0.56	1.53	1.50	-2.00	-2.21	-0.82	3.47	0.62	-2.28	1.61
Hummel	bumblebee	B	5.83	1.74	1.44	1.74	-0.32	-0.21	-1.50	2.65	1.15	1.32	3.21	-0.88	1.11	3.75
Igel	hedgehog	B	5.76	1.86	1.68	1.71	-1.25	-1.43	-2.00	2.25	1.21	0.14	3.54	-2.64	1.09	2.01
Insekt	insect	B	5.19	1.15	1.64	1.45	-1.03	-1.30	-2.33	2.45	-0.21	-0.21	2.80	-2.39	0.04	0.40
Instinkt	instinct	D	3.43	2.18	1.67	1.24	1.91	1.73	0.91	1.94	0.48	1.30	3.50	2.47	0.48	6.76
Jagdhund	hound	B	3.53	1.09	1.38	1.28	1.31	2.09	1.25	2.78	1.66	2.34	2.38	2.40	1.52	6.86
Jähzorn	irascibility	D	3.91	2.38	2.44	2.16	1.31	1.09	1.09	1.19	0.00	0.25	4.99	1.48	-0.03	6.50
Kaffee	coffee	C	8.20	1.94	1.84	1.00	0.78	1.78	0.31	-0.41	-1.34	0.84	3.36	1.74	-1.33	3.96
Kakao	hot choclate	C	6.00	2.21	2.43	1.29	-0.71	0.68	-0.07	-1.04	-1.79	-0.25	4.35	-0.30	-1.78	2.20
Kämpfer	fighter	A	7.86	1.12	1.06	1.00	2.24	2.71	2.12	2.59	2.24	2.56	1.90	3.61	1.92	8.05
Kampfgeist	morale	D	6.11	2.15	1.82	1.45	2.64	2.67	2.42	2.09	1.70	2.58	3.76	3.85	1.43	9.66
Karpfen	carp	B	4.81	0.88	0.85	1.27	-0.88	-0.18	0.42	2.24	0.36	-0.42	1.66	-1.20	0.44	0.80
Karte	card	C	8.95	1.67	1.27	1.03	1.00	0.15	0.24	-2.12	-2.15	1.91	2.57	0.38	-2.26	1.15
Käse	cheese	C	6.89	1.72	1.69	0.97	-1.06	0.13	-0.25	-1.44	-1.91	-0.56	3.01	-1.05	-1.95	-0.13
Kasten	box	C	7.32	1.07	0.71	0.68	-0.82	0.25	0.46	-2.36	-2.64	-0.57	1.32	-0.76	-2.67	-2.25
Kater	tomcat	B	6.64	1.76	1.82	1.79	0.58	0.91	-0.09	2.15	1.39	0.39	3.65	0.79	1.21	5.74
Kätzchen	kitten	B	4.90	2.15	2.45	2.45	-1.73	-1.91	-2.61	2.67	0.61	-0.76	5.01	-3.42	0.70	2.10
Katze	cat	B	7.55	1.53	1.84	1.95	0.16	0.34	-0.61	2.34	1.53	1.32	3.59	-0.03	1.34	5.22
Kissen	pillow	C	6.25	2.26	2.74	1.58	-0.92	-0.79	0.34	-1.63	-2.42	-0.53	4.88	-1.79	-2.37	0.60
Kleidung	clothes	C	8.11	2.03	2.21	1.94	0.27	0.45	0.33	-0.30	-0.79	1.09	4.36	0.17	-0.89	3.90
Knoten	knot	C	6.83	1.29	1.61	0.93	1.29	1.82	0.54	-1.57	-1.54	1.36	2.57	2.12	-1.69	3.32
Köchin	chef	A	5.24	1.88	1.78	0.84	0.84	1.25	0.13	2.59	2.47	1.97	3.15	1.29	2.10	7.02
Kochtopf	pot	C	4.62	1.53	1.16	0.88	-0.69	0.31	0.31	-2.59	-2.50	0.53	2.25	-0.61	-2.61	-0.84
Koffer	suitcase	C	7.78	1.39	1.15	1.06	-0.70	0.09	1.27	-2.30	-2.39	0.39	2.25	-0.80	-2.47	-0.94
König	king	A	8.83	1.16	0.95	0.97	2.45	1.89	1.11	1.87	1.87	1.68	1.80	2.99	1.52	6.71
Krähe	crow	B	4.14	1.03	1.33	1.48	0.33	0.76	0.12	2.39	1.18	0.79	2.40	0.49	1.09	4.17
Küken	chick	B	5.86	2.03	2.09	2.15	-2.29	-2.38	-2.76	2.85	1.12	-0.85	4.35	-4.25	1.12	1.02
Künstler	artist	A	9.29	1.52	1.15	1.12	0.36	0.48	0.48	2.27	2.09	0.15	2.38	0.26	1.76	4.43
Lampe	light	C	6.44	2.15	1.85	1.15	-0.18	0.33	-0.76	-1.79	-2.39	0.12	3.62	-0.27	-2.38	0.99
Landwirt	farmer	A	6.86	1.64	1.03	0.55	0.42	1.85	0.94	2.67	2.48	1.85	2.01	1.57	2.13	6.15
Laubfrosch	tree-frog	B	2.71	1.21	1.15	1.33	-1.30	-1.18	-2.09	1.94	1.30	-0.48	2.27	-2.46	1.10	0.79
Läufer	runner	A	7.35	1.91	1.48	1.24	0.33	1.88	1.21	2.48	1.97	2.30	3.10	1.54	1.70	6.90
Lehrer	teacher	A	9.25	1.14	1.29	0.21	1.43	0.89	0.57	1.54	2.29	1.79	1.70	1.36	1.77	5.27
Liebe	love	D	9.37	2.64	2.75	2.93	2.68	2.54	2.07	2.61	1.32	0.89	5.97	3.76	1.23	11.17
Löffel	spoon	C	5.82	1.09	1.15	0.55	-1.06	-0.12	-1.00	-2.94	-2.64	-1.18	1.72	-1.29	-2.77	-2.63
Mädchen	girl	A	9.78	1.58	1.64	1.88	-0.33	-0.61	-1.12	2.70	2.30	0.52	3.37	-1.25	2.00	4.24

Table A1 continued

noun	translation	cond	logfreq	VAL2	PLE2	APP2	POW	STR	SIZ	ANI	CON	AIM	EVA	POT	HUM	ACT
Marder	marten	B	5.01	1.09	1.63	1.19	0.63	0.28	-1.34	2.41	1.19	1.06	2.59	0.22	1.10	4.15
Marktfrau	market-woman	A	3.04	1.32	1.15	0.97	0.03	0.94	-0.03	2.59	2.24	1.82	2.14	0.45	1.92	4.94
Masseur	masseur	A	4.87	2.26	2.47	0.97	0.38	1.74	0.50	2.21	2.18	2.32	4.25	1.43	1.81	8.05
Maultier	mule	B	3.33	1.24	0.88	0.85	-0.52	0.97	0.79	1.97	1.39	-0.27	1.72	0.12	1.17	2.95
Maulwurf	mole	B	4.85	0.88	0.88	1.31	-1.00	-0.78	-2.13	2.16	1.34	0.47	1.70	-1.88	1.17	1.10
Maurer	bricklayer	A	7.24	1.36	0.79	0.68	0.36	1.82	1.21	1.96	1.89	1.29	1.61	1.50	1.55	4.98
Mehlwurm	mealworm	B	0.00	1.82	2.43	2.61	-1.89	-2.11	-2.61	1.61	-0.36	-1.43	4.81	-3.72	-0.22	0.53
Meister	master	A	9.38	1.91	1.45	0.73	2.39	2.18	1.73	2.03	2.18	2.45	2.77	3.23	1.78	8.37
Messer	knife	C	7.99	0.97	0.97	0.76	1.52	1.91	0.06	-2.27	-2.27	0.58	1.55	2.35	-2.38	1.66
Milchkuh	dairy cow	B	3.09	1.76	1.18	0.97	-0.18	1.05	2.03	1.66	0.08	-0.76	2.52	0.44	0.12	2.89
Mörder	murderer	A	7.84	2.59	2.72	1.66	1.44	1.09	0.38	1.19	1.47	1.78	5.16	1.55	1.09	8.23
Mutter	mother	A	10.17	2.47	2.29	1.76	1.50	1.82	0.38	2.68	2.71	2.15	4.68	2.27	2.30	9.76
Nagel	nail	C	7.62	1.03	0.72	0.78	0.13	0.75	-1.78	-2.69	-2.22	1.13	1.35	0.30	-2.41	-0.49
Natter	viper	B	3.74	0.89	1.47	1.24	-0.13	-0.16	-0.76	1.68	0.61	0.55	2.30	-0.69	0.52	2.27
Nonne	nun	A	5.53	1.50	1.09	0.62	-0.65	-0.35	-0.38	1.50	2.59	1.94	2.01	-1.21	2.00	3.26
Oma	grandmother	A	7.20	2.18	2.27	1.03	0.64	-0.24	-1.09	1.70	2.48	1.15	4.01	-0.25	1.95	5.99
Onkel	uncle	A	7.30	1.61	1.64	0.70	0.45	1.03	1.03	2.06	2.12	0.82	2.71	0.84	1.74	5.49
Opa	grandfather	A	6.46	1.95	1.84	0.82	0.32	-0.11	0.05	1.34	2.00	0.61	3.26	-0.32	1.52	4.60
Panik	panic	D	7.95	2.21	2.48	1.79	1.45	1.27	2.00	1.73	-0.18	-0.36	4.70	1.76	-0.07	6.30
Papier	paper	C	9.08	1.39	1.06	0.97	0.00	-0.30	-0.64	-1.48	-2.00	-0.55	2.10	-0.73	-2.03	-0.79
Parfüm	parfume	C	5.81	1.37	1.82	1.61	0.63	0.24	-0.82	-0.18	-1.21	0.68	3.24	0.19	-1.19	2.40
Pavian	baboon	B	2.30	0.76	0.79	1.26	0.03	1.12	0.41	2.59	1.24	0.32	1.51	0.62	1.16	3.37
Pfarrer	pastor	A	8.01	1.06	0.94	0.38	0.76	0.38	0.12	1.91	2.53	1.94	1.38	0.43	2.03	4.30
Pfleger	nurse	A	6.49	2.31	1.66	0.78	0.94	1.22	0.72	2.53	2.72	2.00	3.32	1.33	2.28	7.42
Pottwal	spermwhale	B	3.22	1.56	1.25	1.53	1.88	2.44	2.88	2.47	1.94	0.97	2.75	3.14	1.68	7.80
Priester	priest	A	7.34	1.09	1.16	0.53	0.50	-0.19	-0.22	1.44	2.25	1.59	1.71	-0.28	1.73	3.55
Psyche	psyche	D	6.22	1.36	1.09	1.12	2.00	1.73	1.15	2.18	1.79	1.48	2.20	2.53	1.51	6.60
Putzfrau	cleaning lady	A	5.81	1.29	1.18	0.92	-0.97	-0.13	-0.97	1.84	1.82	1.00	2.12	-1.24	1.47	2.59
Ratte	rat	B	5.08	1.68	2.13	2.05	0.21	0.00	-1.21	2.32	0.82	0.37	4.08	-0.32	0.80	4.64
Raubtier	predator	B	5.14	0.95	1.13	1.76	2.16	2.55	2.00	2.47	1.47	2.24	2.29	3.42	1.33	7.57
Raupe	caterpillar	B	4.42	0.85	1.33	1.24	-1.48	-1.21	-2.48	2.61	0.24	0.58	2.12	-2.61	0.41	0.06
Redner	speaker	A	7.09	0.92	1.03	0.61	1.32	0.79	0.66	1.63	2.26	2.13	1.49	1.19	1.77	4.97
Rentier	reindeer	B	3.91	1.47	1.39	1.68	0.16	1.34	1.42	2.37	1.08	0.55	2.93	0.93	1.01	5.00
Rentner	pensioner	A	8.19	0.97	0.88	0.55	-0.61	-1.00	-0.58	1.73	2.27	0.36	1.34	-1.78	1.80	1.44
Respekt	respect	D	8.65	2.36	2.04	1.43	2.04	1.89	1.61	0.82	1.39	1.50	4.13	2.72	0.97	8.18
Richter	judge	A	9.76	1.26	1.29	0.58	2.53	2.03	1.32	1.58	2.39	2.47	2.01	3.17	1.86	7.64
Rotfuchs	red fox	B	0.69	1.03	1.06	1.41	0.00	0.65	-0.97	2.59	1.74	1.21	2.07	0.14	1.54	4.04
Schlange	snake	B	7.43	1.25	1.86	1.64	1.36	1.79	0.43	2.54	1.29	1.82	3.22	2.13	1.19	6.98
Schnecke	snail	B	5.12	0.84	1.25	1.59	-2.16	-1.91	-2.63	1.81	0.66	-0.16	2.24	-3.71	0.59	-0.93
Schönheit	beauty	D	7.86	2.06	2.24	2.61	2.03	1.64	0.85	1.67	0.24	0.30	4.80	2.46	0.24	7.58
Schüler	student	A	9.85	1.39	0.85	0.61	-0.94	-0.24	-0.73	2.39	2.30	0.61	1.67	-1.32	1.94	2.44
Schwager	brother in law	A	6.03	1.15	1.00	0.41	0.03	0.18	0.21	1.97	2.00	0.56	1.52	-0.25	1.63	3.04
Schwalbe	swallow	B	5.39	1.26	1.42	1.55	-0.92	-1.00	-1.87	2.47	0.74	0.97	2.72	-2.02	0.77	1.69
Schwester	sister	A	8.61	2.07	2.00	1.75	0.64	0.93	-0.14	2.64	2.50	1.11	4.06	0.85	2.14	7.31
Seele	soul	D	8.19	1.91	1.82	1.55	1.55	1.45	1.39	1.82	1.39	1.06	3.63	1.98	1.15	7.00
Segen	blessing	D	7.26	1.69	1.78	1.38	0.59	0.38	0.44	-0.66	-0.59	0.88	3.32	0.32	-0.81	3.04
Sittich	parakeet	B	2.08	1.00	1.21	1.29	-1.61	-1.61	-2.18	2.16	0.24	-0.82	2.14	-3.05	0.33	-0.78
Skorpion	scorpion	B	4.36	1.26	1.79	1.32	1.56	1.71	-1.15	2.32	1.44	1.21	2.98	2.17	1.27	6.70
Spieler	player	A	10.37	1.00	1.00	0.54	-0.07	0.14	0.32	2.07	2.11	1.25	1.48	-0.35	1.73	3.16
Spinne	spider	B	5.41	1.61	2.03	1.91	0.00	-0.33	-1.36	1.76	0.88	0.52	3.83	-0.77	0.74	3.92
Spitzmaus	shrew mouse	B	1.10	1.21	1.29	1.14	-1.68	-1.93	-2.61	2.57	0.96	0.64	2.30	-3.41	0.96	0.00
Sportler	athlete	A	8.24	1.45	1.45	1.34	0.74	2.24	1.66	2.71	2.24	2.50	2.76	2.15	1.95	7.46
Stinktier	skunk	B	2.64	1.09	1.79	1.33	0.09	-0.09	-0.82	2.21	1.21	0.39	2.84	-0.48	1.08	3.54
Streber	nerd	A	4.20	0.87	1.32	1.11	-0.45	-0.53	-0.39	0.79	1.84	2.45	2.04	-1.24	1.30	2.69
Tante	aunt	A	7.11	1.45	1.34	1.00	-0.05	0.13	-0.34	1.79	1.58	0.18	2.45	-0.36	1.28	3.42
Thunfisch	tuna	B	4.26	1.37	1.29	1.00	-0.97	-0.03	0.82	1.74	0.18	-0.29	2.34	-1.11	0.21	1.37
Tiger	tiger	B	7.47	1.27	1.09	2.30	2.24	2.61	1.94	2.85	1.64	1.79	2.81	3.52	1.52	8.28
Tischler	carpenter	A	5.15	1.54	1.32	0.43	0.50	1.93	0.96	2.32	2.39	2.00	2.17	1.69	2.00	6.34
Tochter	daughter	A	9.78	1.79	1.68	1.71	-0.06	-0.18	-0.65	2.74	2.59	0.59	3.48	-0.66	2.22	5.19
Trainer	coach	A	10.63	1.54	1.14	0.61	1.43	1.75	1.11	2.46	2.43	2.36	2.08	2.16	2.05	6.86
Triumph	triumph	D	8.05	2.00	2.24	1.67	2.33	2.12	1.91	0.85	0.15	2.00	4.21	3.14	0.03	7.86
Truthahn	gobbler	B	4.34	0.92	1.16	1.68	-0.79	0.26	0.79	2.29	0.74	-0.66	2.25	-0.72	0.73	2.11
Turner	gymnast	A	6.71	1.59	1.06	1.25	0.22	2.16	0.06	2.56	2.50	2.19	2.42	1.69	2.12	6.76
Uhu	eagle owl	B	4.70	1.29	1.26	1.71	0.47	0.50	0.34	1.92	1.13	0.82	2.66	0.35	0.97	4.17
Unmut	discontent	D	7.88	2.11	2.08	1.50	-0.05	-0.21	0.00	-0.74	-0.21	-0.79	4.02	-0.67	-0.53	2.63
Unschuld	innocence	D	7.36	1.69	1.50	1.44	0.25	0.34	-0.03	0.44	0.28	0.28	3.06	0.05	0.05	3.22
Vater	father	A	9.98	2.13	2.08	1.08	1.45	1.50	1.45	1.82	1.97	1.50	3.80	1.95	1.59	7.70
Vetter	cousin	A	6.38	1.27	1.21	0.55	0.15	0.42	0.67	2.00	2.12	0.58	1.92	0.07	1.73	3.86
Vogel	bird	B	7.95	1.52	1.42	1.82	-0.52	-0.48	-0.97	2.76	1.91	0.73	3.07	-1.26	1.71	3.69
Vorteil	advantage	D	8.72	2.15	2.18	1.36	1.85	1.76	1.24	-0.67	-0.55	1.79	4.09	2.46	-0.77	6.20
Wagen	vehicle	C	9.32	1.16	1.25	1.09	0.72	1.25	0.88	-1.69	-2.06	0.88	2.19	1.22	-2.11	1.50
Walross	walrus	B	2.48	0.87	0.89	1.34	1.42	2.11	2.42	1.82	0.95	-0.11	1.74	2.51	0.81	5.03
Wasser	water	C	10.06	2.36	2.36	2.04	2.64	2.32	1.36	2.11	-1.11	1.21	4.82	3.52	-0.70	7.93
Wespe	wasp	B	4.58	1.27	2.15	1.24	0.64	0.03	-1.67	2.52	0.06	1.00	3.31	-0.01	0.26	3.80
Whiskey	whiskey	C	4.94	1.36	1.68	1.25	0.54	1.96	-0.25	-1.29	-1.79	-0.39	2.88	1.73	-1.83	2.69
Widder	ram	B	6.03	1.00	0.79	0.97	0.91	1.85	1.06	2.36	1.73	0.76	1.51	1.90	1.50	5.09
Wiesel	weasel	B	4.45	0.91	0.85	0.85	-0.33	0.15	-0.82	2.55	1.06	0.88	1.43	-0.54	1.02	2.12
Wildschwein	boar	B	5.06	0.69	1.16	1.38	1.59	2.03	1.53	2.63	1.38	0.69	1.89	2.54	1.28	5.88
Winzer	vintner	A	6.60	1.47	1.32	0.50	0.50	1.08	0.50	2.00	2.03	1.92	2.16	0.90	1.66	5.18
Wissen	knowledge	D	10.25	2.61	2.30	2.12	2.85	2.52	2.09	1.12	2.00	2.03	5.01	3.85	1.48	10.83
Witwe	widow	A	7.15	0.93	1.11	0.86	-1.39	-0.79	-0.93	0.11	2.07	-0.82	1.72	-2.13	1.35	0.75
Witwer	widower	A	4.77	1.24	1.27	0.67	-1.33	-0.88	-0.15	0.00	1.27	-0.45	2.03	-2.16	0.73	0.49
Wohlstand	prosperity	D	7.33	1.82	2.15	1.44	2.21	1.56	1.44	-0.59	-0.68	0.91	3.84	2.52	-0.86	5.73
Wurzel	root	C	6.16	1.42	0.82	1.39	1.67	2.27	1.24	1.39	-0.67	0.97	2.11	2.81	-0.50	4.66
Zahnarzt	dentist	A	6.51	1.62	1.68	0.59	1.09	0.91	0.41	1.88	2.03	2.21	2.70	1.15	1.64	6.01
Ziege	goat	B	5.76	0.88	0.82	1.03	-0.73	-0.12	-0.24	1.97	1.00	-0.48	1.48	-1.05	0.87	1.19
		mean	6.36	1.57	1.55	1.31	0.43	0.65	0.15	1.38	0.86	0.92	2.95	0.46	0.66	4.29
		sd	2.15	0.51	0.54	0.55	1.24	1.23	1.29	1.50	1.48	1.05	1.14	1.94	1.36	3.08
		min	0.00	0.69	0.71	0.21	-2.29	-2.38	-2.76	-2.94	-2.64	-1.43	1.32	-4.25	-2.77	-3.17
		max	10.63	2.91	2.85	2.93	2.85	2.71	2.88	2.88	2.75	2.66	6.01	3.85	2.30	11.17

Table A2 Means, standard deviations (sd), minimum (min) and maximum (max) ratings and factor values per noun type (humans, animals, concrete things and abstract things).

		VAL	PLE	APP	POW	STR	SIZ	ANI	CON	GOA	EVA	POT	HUM	ACT
humans	mean	1.61	1.47	0.95	0.57	0.72	0.30	1.99	2.22	1.53	3.33	1.06	2.04	6.81
	sd	0.45	0.46	0.47	1.03	1.07	0.79	0.60	0.34	0.91	0.98	1.60	0.33	2.39
	min	0.87	0.79	0.21	-2.06	-2.11	-1.79	0.00	1.27	-1.00	1.99	-3.28	0.97	1.19
	max	2.68	2.82	2.36	2.53	2.71	2.12	2.74	2.75	2.65	6.39	4.05	2.54	12.17
animals	mean	1.23	1.37	1.50	-0.23	0.08	-0.58	2.31	1.03	0.38	3.25	-0.09	1.20	4.45
	sd	0.36	0.43	0.41	1.22	1.40	1.58	0.37	0.59	0.95	0.85	2.11	0.48	2.67
	min	0.69	0.76	0.85	-2.29	-2.38	-2.76	1.30	-0.36	-1.43	2.08	-3.81	0.02	0.40
	max	2.15	2.45	2.61	2.36	2.61	2.88	2.88	2.25	2.34	5.66	3.96	2.23	10.50
concrete things	mean	1.63	1.56	1.19	0.22	0.78	0.15	-1.40	-1.92	0.42	3.58	0.86	-1.71	2.83
	sd	0.48	0.60	0.43	1.10	1.01	0.93	1.26	0.61	0.92	1.18	1.61	0.67	2.74
	min	0.97	0.71	0.55	-1.32	-0.79	-1.78	-2.94	-2.64	-1.18	1.97	-1.55	-2.53	-1.84
	max	2.74	2.76	2.38	2.64	2.71	1.64	2.11	-0.32	1.91	6.39	4.28	-0.08	9.41
abstract things	mean	2.14	2.08	1.76	1.71	1.56	1.34	0.94	0.49	1.25	4.85	2.63	0.54	8.32
	sd	0.39	0.51	0.56	0.70	0.67	0.61	0.98	0.76	0.89	1.09	1.09	0.70	2.03
	min	1.36	1.00	0.79	-0.05	-0.21	-0.03	-0.74	-0.82	-0.79	2.60	-0.23	-0.62	3.96
	max	2.91	2.85	2.93	2.85	2.67	2.42	2.61	2.00	2.66	6.66	4.29	1.75	12.50

Table A3: Mean ratings and standard deviations of actor-ratings in order to test the construct validation of the model.

condition	mean	sd
human	3.11	0.95
animal	2.48	0.98
concrete	2.09	1.04
abstract	2.69	1.04

Supplementary Material B: Summary of the best-fitting structural equation model for the results of Experiment 1

The best-fitting linear mixed effects model of the data for human nouns only is summarized in Tables C1 (fixed effects) and C2 (random effects).

Table C1: Summary of results (fixed effects) of the Markov chain Monte Carlo sampling (MCMC) for human nouns only in order to evaluate p-values from posterior distributions for the best-fitting model with random slope but without random correlation parameters. Besides model estimates, MCMC mean and corresponding p-values, the highest posterior distribution (HPD) 95% confidence interval with the lower (HPD95lower) and upper (HPD95upper) bound of the degrees of freedom is reported.

	Estimate	MCMCmean	HPD95lower	HPD95upper	pMCMC	Pr(>|t|)
(Intercept)	-0.6720	-0.6645	-1.9567	0.6525	0.3122	0.3122
O-S	-1.0050	-1.0081	-1.3514	-0.6420	0.0001	0.0000
ROIright-post	-0.6324	-0.6339	-1.2929	0.0431	0.0642	0.0674
ROIleft-ant	0.6375	0.6398	-0.0471	1.2889	0.0652	0.0652
ROIright-ant	-0.2310	-0.2297	-0.9250	0.4234	0.5116	0.5041
O-S:ROIback-right	0.1761	0.1754	-0.0307	0.3822	0.0952	0.0945
O-S:ROIfront-left	-0.2347	-0.2348	-0.4287	-0.0159	0.0274	0.0259
O-S:ROIfront-right	-0.0600	-0.0607	-0.2730	0.1356	0.5534	0.5687
S-O:FRQ	0.1968	0.1957	-0.0042	0.3876	0.0536	0.0480
O-S:freq.log	0.3914	0.3911	0.1908	0.5855	0.0004	0.0001
ROIright-post:freq.log	-0.0380	-0.0376	-0.1445	0.0701	0.4912	0.4908
ROIleft-ant:freq.log	-0.1899	-0.1899	-0.2944	-0.0780	0.0004	0.0006
ROIright-ant:freq.log	-0.3558	-0.3561	-0.4642	-0.2483	0.0001	0.0000
FRQ:ACT	0.0127	0.0127	-0.0015	0.0268	0.0780	0.0756
S-O:case	0.4618	0.4560	-1.1493	1.9860	0.5628	0.5581
O-S:case	0.2059	0.1983	-1.3202	1.7920	0.7966	0.7939
ROIright-post:case	0.1005	0.1006	-0.7475	1.0149	0.8242	0.8257
ROIleft-ant:case	-0.1885	-0.1948	-1.0862	0.7026	0.6606	0.6797
ROIright-ant:case	-1.4707	-1.4738	-2.3239	-0.5275	0.0018	0.0013
O-S:FRQ:ACT	-0.0074	-0.0074	-0.0118	-0.0030	0.0012	0.0013
ROIright-post:FRQ:ACT	-0.0040	-0.0041	-0.0102	0.0023	0.1992	0.2110
ROIleft-ant:FRQ:ACT	-0.0081	-0.0081	-0.0142	-0.0015	0.0128	0.0123
ROIright-ant:FRQ:ACT	-0.0048	-0.0048	-0.0107	0.0017	0.1372	0.1396
ROIleft-post:FRQ:case	-0.0783	-0.0774	-0.2915	0.1349	0.4668	0.4680
ROIright-post:FRQ:case	-0.0697	-0.0687	-0.2842	0.1467	0.5192	0.5180
ROIleft-ant:FRQ:case	0.0154	0.0173	-0.1998	0.2306	0.8718	0.8861
ROIright-ant:FRQ:case	0.1709	0.1724	-0.0360	0.3963	0.1126	0.1131

Table C2: Summary of results (random effects) of the Markov chain Monte Carlo sampling (MCMC) for human nouns only in order to evaluate p-values from posterior distributions for the best-fitting model with random slope but without random correlation parameters. Besides model estimates, MCMC mean and corresponding p-values, the highest posterior distribution (HPD) 95% confidence interval with the lower (HPD95lower) and upper (HPD95upper) bound of the degrees of freedom is reported.

Groups	Name	Std.Dev.	MCMCmedian	MCMCmean	HPD95lower	HPD95upper
itm	(Intercept)	0.6208	0.6198	0.6269	0.5045	0.7629
subj	ACT	0.2084	0.2134	0.2165	0.1664	0.2656
subj	(Intercept)	1.5332	1.4303	1.4371	1.1755	1.7194
Residual		4.0024	4.0026	4.0026	3.9767	4.0289

Supplementary Material D: ERP analyses for the wh-pronoun position in Experiment 2

Figure D1 shows grand average ERPs at the position of the wh-pronouns in the subject-initial (pronoun “wer”, red trace) and object-initial (pronoun “wen”, blue trace) conditions, respectively. As is apparent from the plot, nominative and accusative wh-pronouns did not lead to differential ERP effects. This impression based on visual inspection was confirmed by a statistical analysis in the 300-500 ms time window (i.e. the only time window showing a very slight visual difference between the two conditions), which revealed neither a significant main effect of word order ($F(1,39) = 1.63$, $p > 0.2$) nor an interaction of word order and ROI ($F < 1$). Thus, the ERP effects at our critical NP2 position cannot be attributed to earlier changes elicited by the different wh-pronouns.

Figure D1: Grand average ERPs at the position of the wh-pronoun in Experiment 2. The plot contrasts the nominative wh-pronoun "wer" in the subject-initial condition (red trace) with the accusative wh-pronoun "wen" in the object-initial condition (blue trace). Negativity is plotted upwards.

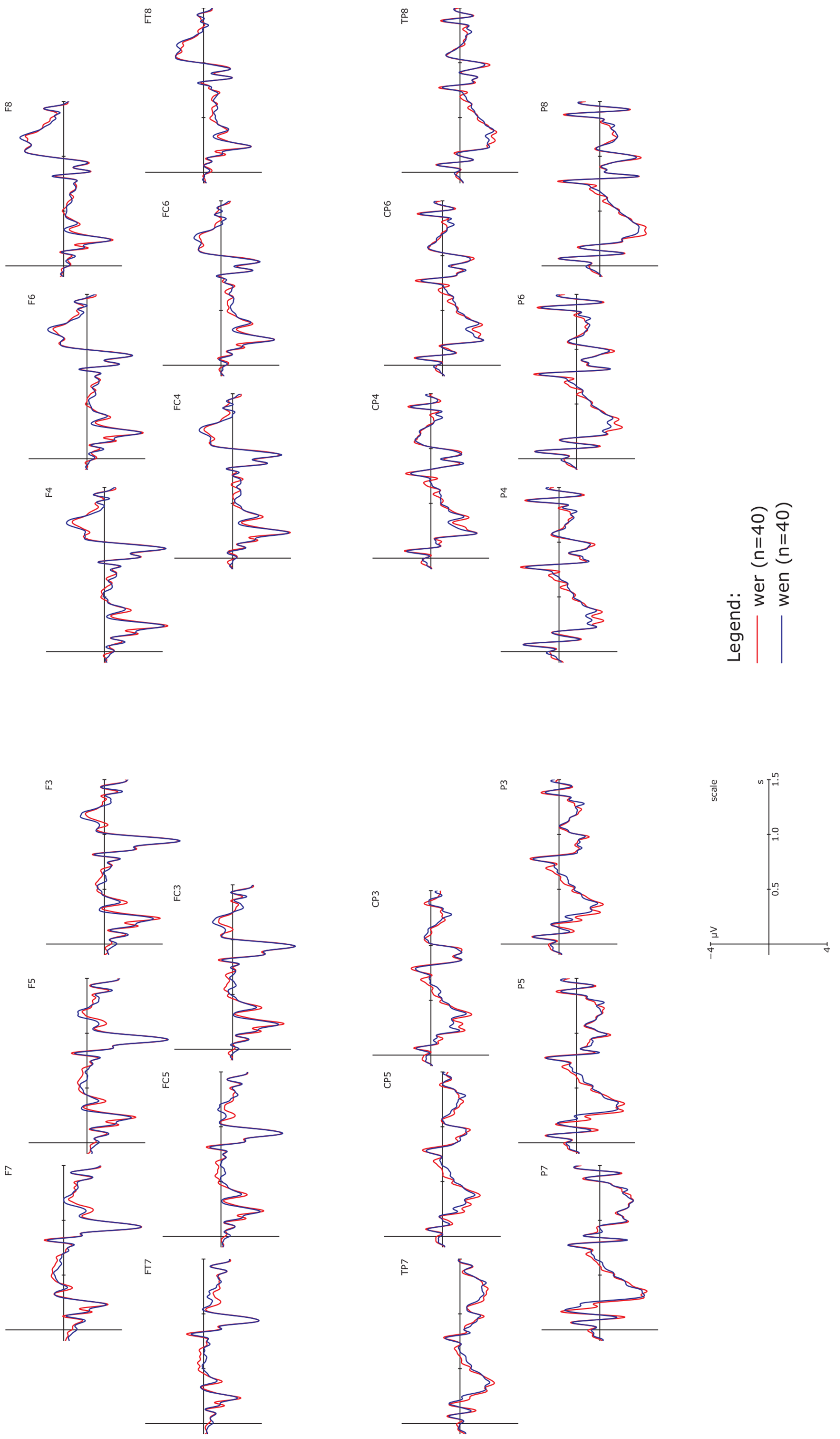

Supplement: Supplementary file 1 [file Data_Sheet_1.PDF]
